# Multiparametric High-Content Cell Painting Identifies Copper Ionophores as Selective Modulators of Esophageal Cancer Phenotypes

**DOI:** 10.1021/acschembio.2c00301

**Published:** 2022-06-13

**Authors:** Rebecca E. Hughes, Richard J. R. Elliott, Xiaodun Li, Alison F. Munro, Ashraff Makda, Roderick N. Carter, Nicholas M. Morton, Kenji Fujihara, Nicholas J. Clemons, Rebecca Fitzgerald, J. Robert O’Neill, Ted Hupp, Neil O. Carragher

**Affiliations:** † Cancer Research UK Edinburgh Centre, Institute of Genetics & Cancer, The University of Edinburgh, Western General Hospital, Edinburgh EH4 2XR, U.K.; ‡ MRC Cancer Unit, Hutchison-MRC Research Centre, University of Cambridge, Cambridge CB2 0XZ, U.K.; § Centre for Clinical Brain Sciences, Chancellors Building, University of Edinburgh, Edinburgh EH16 4SB, U.K.; ∥ Centre for Cardiovascular Science, The Queen’s Medical Research Institute, Edinburgh BioQuarter, Edinburgh EH16 4TJ, U.K.; ⊥ Gastrointestinal Cancer Program, Cancer Research Division, Peter MacCallum Cancer Centre, Melbourne 3000, Victoria, Australia; # Sir Peter MacCallum Department of Oncology, The University of Melbourne, Parkville 3010, Victoria, Australia; ∇ Cambridge Oesophagogastric Centre, Cambridge University Hospitals Foundation Trust, Cambridge CB2 2QQ, U.K.; □ Early Cancer Institute, Hutchison Research Centre, University of Cambridge, Cambridge CB2 0XZ, U.K.

## Abstract

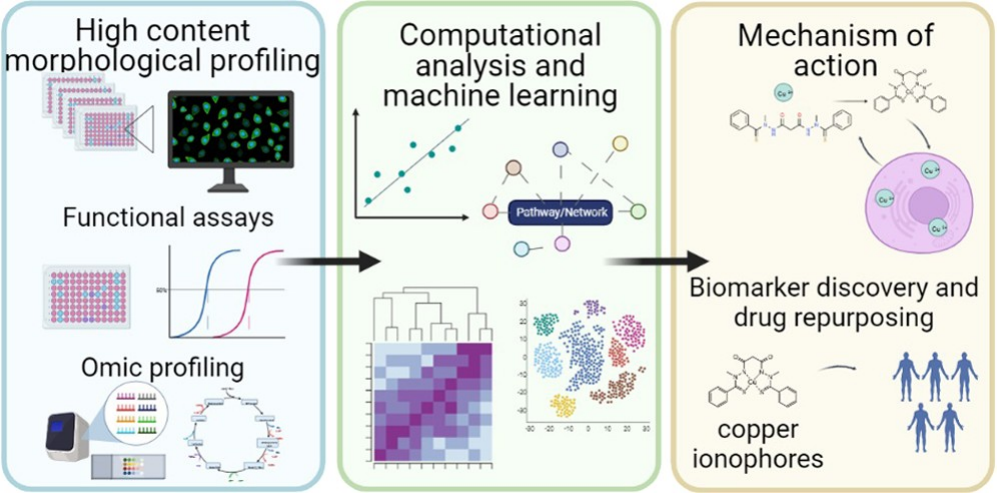

Esophageal adenocarcinoma is of increasing global concern due to increasing incidence, a lack of effective treatments, and poor prognosis. Therapeutic target discovery and clinical trials have been hindered by the heterogeneity of the disease, the lack of “druggable” driver mutations, and the dominance of large-scale genomic rearrangements. We have previously undertaken a comprehensive small-molecule phenotypic screen using the high-content Cell Painting assay to quantify the morphological response to a total of 19,555 small molecules across a panel of genetically distinct human esophageal cell lines to identify new therapeutic targets and small molecules for the treatment of esophageal adenocarcinoma. In this current study, we report for the first time the dose–response validation studies for the 72 screening hits from the target-annotated LOPAC and Prestwick FDA-approved compound libraries and the full list of 51 validated esophageal adenocarcinoma-selective small molecules (71% validation rate). We then focus on the most potent and selective hit molecules, elesclomol, disulfiram, and ammonium pyrrolidinedithiocarbamate. Using a multipronged, multitechnology approach, we uncover a unified mechanism of action and a vulnerability in esophageal adenocarcinoma toward copper-dependent cell death that could be targeted in the future.

## Introduction

Esophageal cancer is emerging as a serious global health care issue due to increasing incidence, a lack of effective treatments, and poor prognosis.^1^ Combined, the two major histological subtypes—esophageal adenocarcinoma (EAC) and esophageal squamous cell carcinoma (ESCC)—represent the sixth leading cause of cancer deaths worldwide with fewer than one in five patients surviving five years from diagnosis.^2^ A shift in epidemiology over the last 50 years has meant the incidence of EAC now vastly exceeds that of ESCC in western countries,^1^ accounting for more than 90% of esophageal cancers in the United States.^3^

Whole genome sequencing of clinical samples has recently highlighted EAC as highly heterogeneous, characterized by frequent large-scale genomic rearrangements and copy number alterations.^4^ Despite recent advances in targeted therapies for tumors expressing HER2, VEGFR2, or PDL1,^5−9^ survival rates remain low for a large proportion of patients and, overall, 5-year survival rates remain less than 20%. This highlights the limitations of modern target-based drug discovery strategies to impact upon complex heterogeneous diseases such as EAC. Phenotypic drug discovery describes the screening and selection of compounds based on quantifiable phenotypic end points without prior knowledge of the drug target.^10,11^ It is therefore an attractive strategy for heterogeneous diseases, where there is a lack of understanding of disease biology and actionable targets.

Cell Painting is a high-content phenotypic screening assay that multiplexes six fluorescent probes labeling multiple cellular compartments. When combined with image analysis and computational biology tools, the Cell Painting assay generates a phenotypic fingerprint for every cell, following chemical or genetic perturbation to identify chemical starting points and novel targets and help guide mechanism-of-action studies.^12,13^ The Cell Painting assay has recently been used to screen 30,616 small-molecule compounds in the U2OS osteosarcoma cell line, generating a large repository of compound phenotypic fingerprints to support the development of new methods, including artificial intelligence/machine learning approaches, which associate cell phenotypes with chemical structures.^14^

In the current study, we have undertaken a comprehensive small-molecule phenotypic screen using the Cell Painting assay to quantify the morphological response to a total of 19,555 small molecules across a panel of six genetically distinct human EAC cell lines and two nontransformed tissue-matched control cells to identify new therapeutic targets and small molecules for the treatment of EAC.^15^ We report for the first time the full list of primary screening hits and dose–response hit validation studies from the target-annotated LOPAC and Prestwick FDA-approved compound libraries. Further, we characterize the most potent and selective hit compounds (elesclomol, disulfiram, and ammonium pyrrolidinedithiocarbamate (PDTC)) in a holistic approach to understand the molecular mechanisms that confer drug sensitivity and potentially identify new therapeutic targets and classes of small molecules for the treatment of EAC.

Previous studies have indicated that these three compounds have varied targets and therefore likely act via multiple distinct mechanisms.^16−22^ Elesclomol has been reported to act as a reactive oxygen species inducer,^22^ while disulfiram is an alcohol dehydrogenase inhibitor,^19^ and ammonium PDTC is a nuclear factor kappa B (NFkB) inhibitor.^16,17^ Disulfiram and ammonium PDTC are also reported to affect the proteasome via multiple mechanisms, including contradictory reports of proteasome inhibition^23^ and NLP4 aggregation^21^—a key component of the p97/VCP segregase essential for protein turnover. Despite differing and at times conflicting reported activities, structurally, the compounds share similarities since all three contain thiocarbonyl groups, and they are known copper chelators. Ammonium PDTC is part of the family of dithiocarbamates, while disulfiram, a thiuram disulfide, breaks down in acidic or Cu(II)-rich environments to produce a dithiocarbamate, diethyldithiocarbamate (DDTC).^24^ DDTC and ammonium PDTC, like other dithiocarbamates, are known to form complexes with transition elements but are most stable in a Cu(II) chelate.^25^ Although elesclomol is structurally unrelated to the dithiocarbamates, it also forms organometallic complexes, particularly with Cu(II), due to its dihydrazide and thiocarbonyl groups.^26,27^

In this work, we employ a multipronged, multitechnology approach and uncover a unified mechanism of action shared by all three compounds. We believe the holistic approach we have employed in this study supports the identification of hit compound mechanisms of action that target complex heterogeneous diseases with high selectivity.

## Results and Discussion

### Comprehensive Small-Molecule Profiling

The aim of this study was to perform an unbiased and target-agnostic phenotypic screen to identify potential drug repurposing opportunities, new chemical starting points, and targets to stimulate drug discovery in the challenging disease area of esophageal cancer. To uncover the mechanism of action of phenotypic hits, which display high selectivity for EAC lines relative to nontransformed tissue-matched controls, we have generated multiparametric cellular phenotypic fingerprints for each compound. For validated compound hits, which display high sensitivity and selectivity for EAC cell lines, we link compounds that share similar phenotypic fingerprints and transcriptomic profiles to the chemical structure to further elucidate the compound mechanism of action. In this work, we began by performing image-based phenotypic profiling of 72 compound hits identified from our primary phenotypic screen^15^ (Supporting Table 1) as dose responses across a panel of heterogeneous esophageal cell lines. The dose–response validation was performed across two phenotypic assay end points: 1. nuclei count to quantify cell survival and 2. multiparametric morphometric response (quantifying 702 features) to provide a more in-depth and unbiased analysis of phenotypic response to compound treatments.

**Figure 1:**
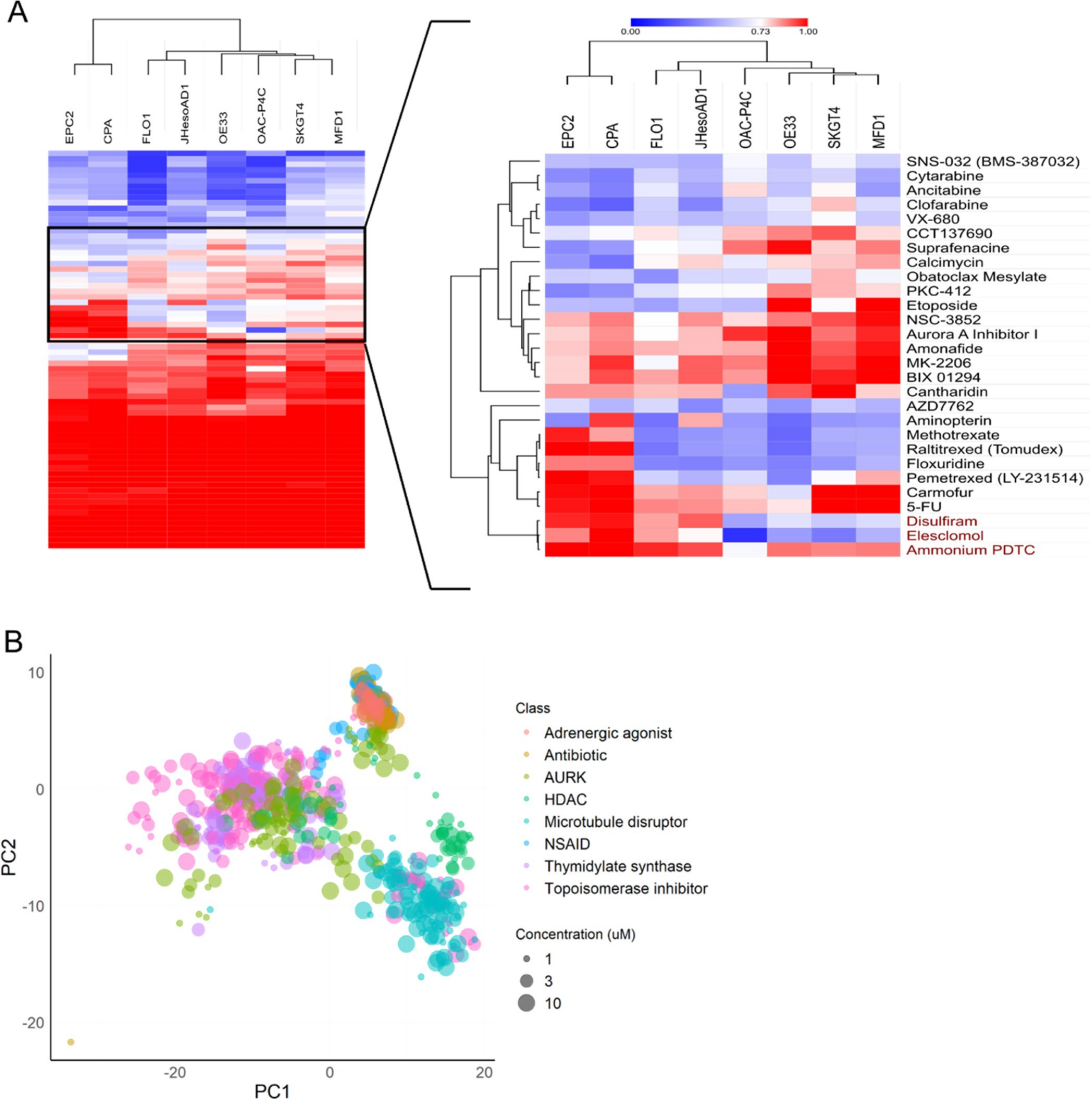
Validation results across 72 compound hits from LOPAC and Prestwick libraries. (A) Univariate analysis of area under the curve (AUC) values across the cell panel for all compounds. Red = higher AUC, less potent; blue = lower AUC, more potent. (B) Phenotypic clustering of compound hits belonging to classes containing two or more compounds.

Testing the 72 compounds for dose-dependent cell survival, we found that 47 of the 72 compounds (65% validation rate) reduced cell survival in a dose-dependent manner, of which 11 (15%) showed strong selectivity for the EAC cells over the tissue-matched control lines (Figure 1A, Supporting Table 2, and Supporting Data). Three of the selective compounds (Aminopterin, AZD7762, and Carmofur) were initially identified as active in the multivariate primary analysis but not the cell survival analysis, demonstrating the utility of morphometric phenotypic screening to detect phenotypically active compounds that would otherwise go undetected from univariate survival analyses of single-point concentrations at the primary screening stage.

In the unbiased phenotypic analysis, we also developed a multivariate morphological dose response to validate morphological changes over a range of concentrations from maximal to minimal effect. Here, 51 of the 72 total compounds (71%) caused dose-dependent phenotypic changes, of which 46 (64%) were selective for the EAC cells over the tissue-matched controls (Supporting Table 2). Across the two end points, we identified 51 small molecules (71% validation rate) that showed dose-dependent activity across two or more of the EAC cell lines.

Using the phenotypic information from such multivariate compound profiles, we can gain a deeper understanding of the compound activity, mechanism of action, cellular response, and selectivity over tissue-matched controls.^28^ We have shown previously, using a reference library of well-annotated compounds (Supporting Table 3), that compounds with a shared mechanism of action cluster phenotypically.^15^ The phenotypic dose responses for the validation hits demonstrate class-specific phenotypic clustering (Figure 1B), suggesting most are acting via their annotated mechanism in the EAC cell lines. However, a few outliers clustered with unexpected classes of compounds, and these warranted further investigation. Known classes identified include aurora kinase inhibitors, dihydrofolate reductase inhibitors, and thymidylate synthase inhibitors.

Of note, several of our hits, including AZD7762 and camptothecin, were also identified among the top hits in an esophageal patient-derived organoid screen,^29^ demonstrating the ability of this high-throughput assay to recapitulate results from low-throughput, expensive, and complex patient-derived organoid assays, emphasizing its relevance to patient tumors and the disease.

### Hit Follow-Up

**Figure 2:**
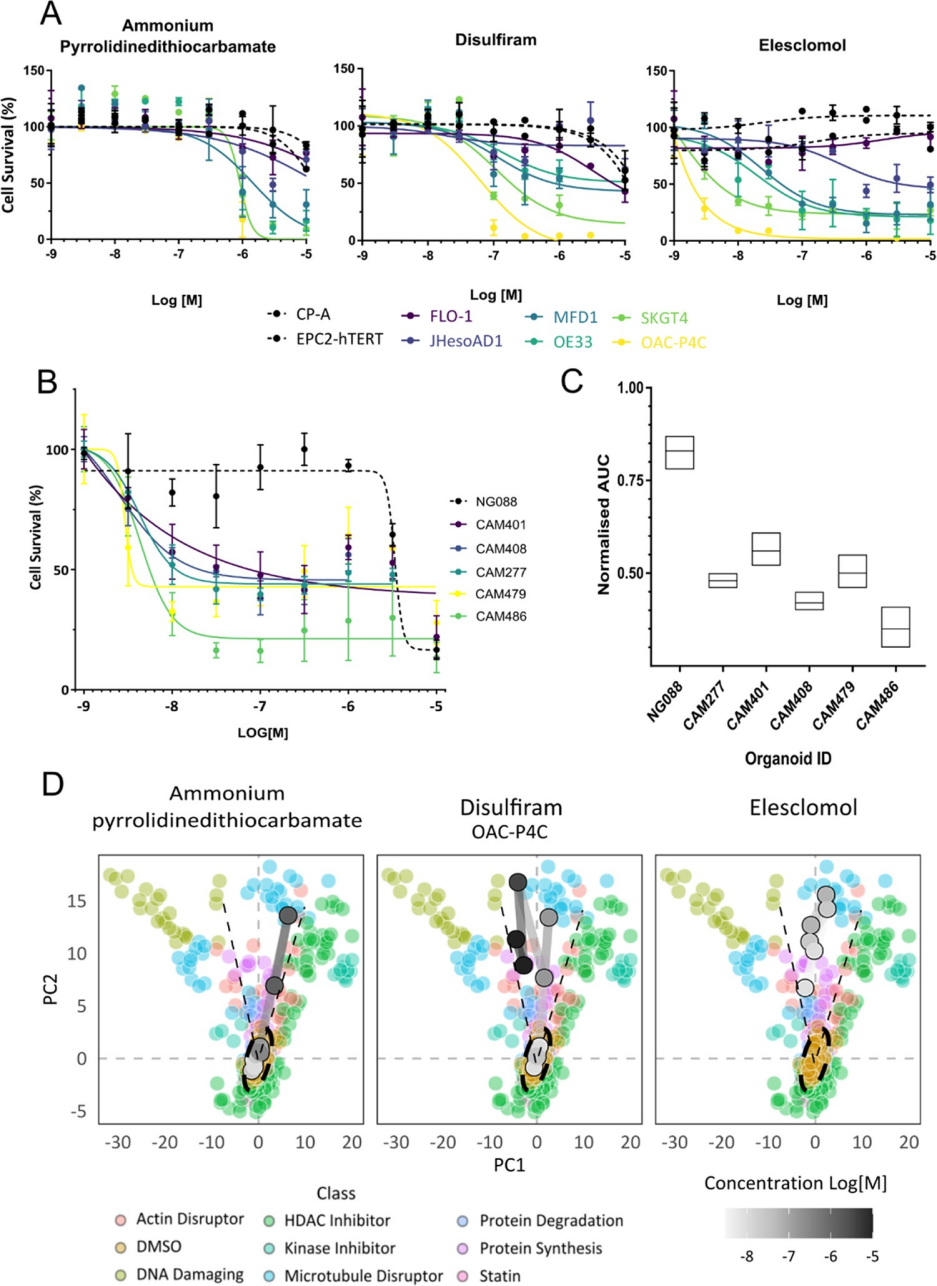
Dose responses for ammonium pyrrolidinedithiocarbamate, disulfiram, and elesclomol. (A) Univariate validation dose responses across the cell panel. (B) Patient-derived organoid dose responses for elesclomol. NG088—normal gastric control organoid, *n* = 3. (C) Comparison of IC_50_ for normal gastric and EAC organoids, *n* = 3, *p* < 0.005. (D) First two principal components of multivariate phenotypic dose responses in OAC-P4C (most sensitive cell line) overlaid on the reference library of compounds. Phenotypic trajectory of dose responses depicted by black dotted line.

We then chose to follow up the most potent and selective small molecules identified across our assay panel— elesclomol, disulfiram, and ammonium PDTC (Figure 1A (red text) and 2A). Elesclomol was the most potent of the compounds, with activity in the low nanomolar range, although activity varied across the panel of genetically distinct EAC cell lines (Figure 2A and Supporting Table 4), followed by disulfiram and then ammonium PDTC. Critically, the dose responses show no toxicity in either of the tissue-matched nontransformed control cell lines (CP-A, a Barrett’s esophagus cell line and EPC2-hTERT, a squamous esophageal cell line) and both continued to proliferate at their normal rate (Figure 2A). The mechanism of EAC cell death is caspase-independent and cannot be rescued by ferroptosis inhibitors (Supporting Figure 1). These data point to a novel form of cell death, cuproptosis, a recently proposed mechanism of cell death specific to copper.^30^

To validate our top hit and confirm its activity in a more complex and disease-relevant assay, we tested elesclomol across a panel of patient-derived organoids, including one normal gastric epithelial and five EAC patient-derived organoids (Figure 2B,C). Elesclomol showed similar potency in the organoids compared to the adherent EAC cell lines, with IC_50_ values in the low nanomolar range (Supporting Table 5). Elesclomol was also highly selective, inhibiting the viability of tumor-derived organoids and not the normal gastric organoid NG088 (Figure 2B,C), demonstrating it to be highly potent and selective for EAC over tissue-matched control cells in both two-dimensional (2D) and three-dimensional (3D) models. Together, these data demonstrate that our morphological, high-content assay is able to identify biologically relevant and highly selective hit molecules.

Using the phenotypic information from the Cell Painting concentration responses, we built multivariate dose responses, tracking cellular morphological changes as a product of compound concentration. We overlaid these dose responses on a library of reference compounds we have published previously^15^ (Supporting Table 3) to better understand how the compounds affect the cells within the context of known drug mechanisms of action. The phenotypic dose responses show that both disulfiram and ammonium PDTC move from phenotypically inactive at low concentrations, clustering with the dimethyl sulfoxide (DMSO), to phenotypically active at higher concentrations (Figure 2D), while elesclomol is active at all concentrations tested. All three compounds cluster away from the known pharmacological classes in the reference library, suggesting a mechanism of action that is distinct from the reference library (Figure 2D). Of note, the compounds do not cause any phenotypic changes in the CP-A or the EPC2-hTERT cell lines across the dose responses (Supporting Figure 2), further demonstrating strong selectivity.

### Shared Mechanism of Action

**Figure 3:**
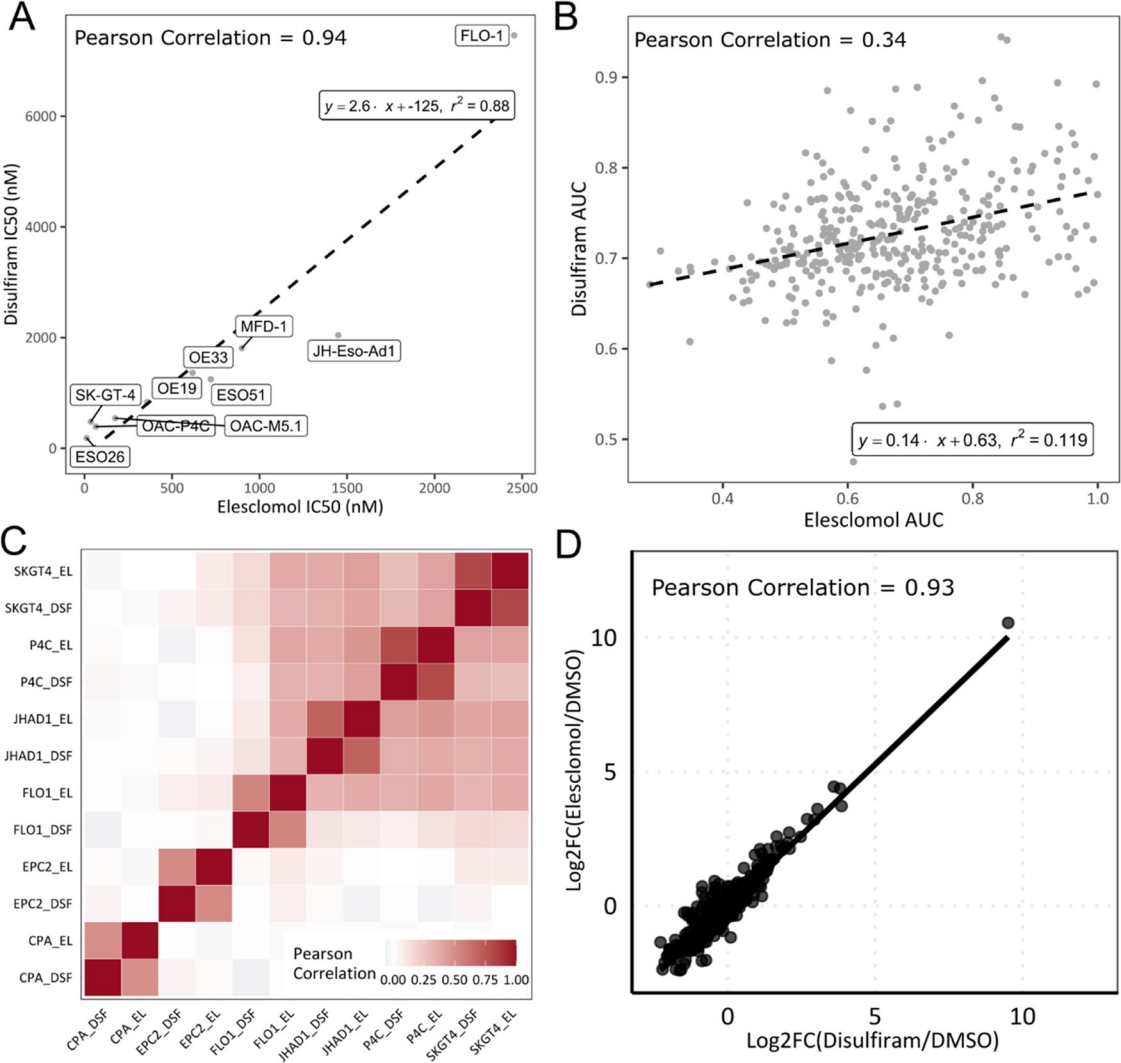
Correlations between disulfiram and elesclomol. (A) IC_50_s across the expanded EAC cell panel using CellTitre-Glo (Promega). (B) Pan cancer correlation of area under the curve. Data from DepMap PRISM Repurposing Secondary Screen 19Q4, *n* = 373. (C) Correlation heat map of log 2 fold gene expression changes across both EAC and tissue-matched controls using the NanoString nCounter platform. (D) Correlation of average gene expression changes across the OAC-P4C cell line. *n* = 3. DSF–Disulfiram, EL–Elesclomol.

Previous studies have indicated that these three compounds have varied targets and therefore likely act via multiple distinct mechanisms.^16−22^ However, our data indicate that all three compounds belong to the same morphological cluster. Quantification via correlation of the multivariate phenotypic signatures confirmed all three compounds induce a shared morphological response (Pearson correlation 0.61 and 0.81 within OAC-P4C and SK-GT-4 cells, respectively) (Supporting Figure 3 for OAC-P4C images), suggesting a potentially shared mechanism in EAC. Furthermore, studying the univariate cell survival analysis, the compounds also shared the same sensitivity profile across the panel of cell lines, with OAC-P4C showing the greatest sensitivity and FLO-1 showing the weakest (Figures 2A,3A). We quantified the sensitivity profile by Pearson correlation of IC_50_ values for disulfiram and elesclomol across an expanded panel of EAC cell lines, which confirmed a shared sensitivity profile across EAC cell lines (Pearson 0.94, *p*-value < 0.001) (Figure 3A). Similar analyses using data across 373 cancer cell lines from multiple tumor types from the Cancer Dependency Map (DepMap)^31,32^ also demonstrated a significant correlation (Pearson 0.34, *p*-value < 0.001) (Figure 3B). Combined, these data suggest that the compounds act through a shared mechanism in OAC.

To further confirm a shared mechanism, we examined compound-induced gene expression signatures. Comparison of signatures can be used to discover new connections among compounds as well as to identify targets and mechanisms of action in a more complex setting than single-gene contributions.^33,34^ We used the NanoString nCounter platform to quantify and compare transcript expression following treatment with elesclomol or disulfiram. A correlation heat map of cell lines and treatments showed that the compound-induced signatures are very similar within a given cell line and to a lesser extent across cell lines (Figure 3C). Second, the EAC lines formed a distinct cluster to the tissue-matched control lines (CP-A and EPC2-hTERT) (Figure 3C). Pearson correlation of the elesclomol- and disulfiram-induced gene expression signatures quantitatively confirmed there was a very strong relationship between the two treatments (Figure 3D and Supporting Figure 4), demonstrating that disulfiram and elesclomol induce the same gene expression signature. Interestingly, neither elesclomol nor disulfiram induced any significant gene expression changes in the two tissue-matched controls (Supporting Figure 5).

### Mechanism Deconvolution

Disulfiram is a well-defined ALDH inhibitor; to rule out this as the mechanism in EAC cells, three ALDH inhibitors were tested for cytotoxicity across esophageal cell lines. CVT-10216, a potent and selective reversible inhibitor of ALDH2 (mitochondrial ALDH), and two potent ALDH1A1 inhibitors (NCT-501 and A37) were tested, none of which had any effect on cell viability (up to 10 uM) in either the tissue-matched control or EAC cell lines (Supporting Figure 6). We therefore do not believe that disulfiram, elesclomol, and ammonium PDTC are targeting the sensitive EAC lines via ALDH inhibition.

**Figure 4:**
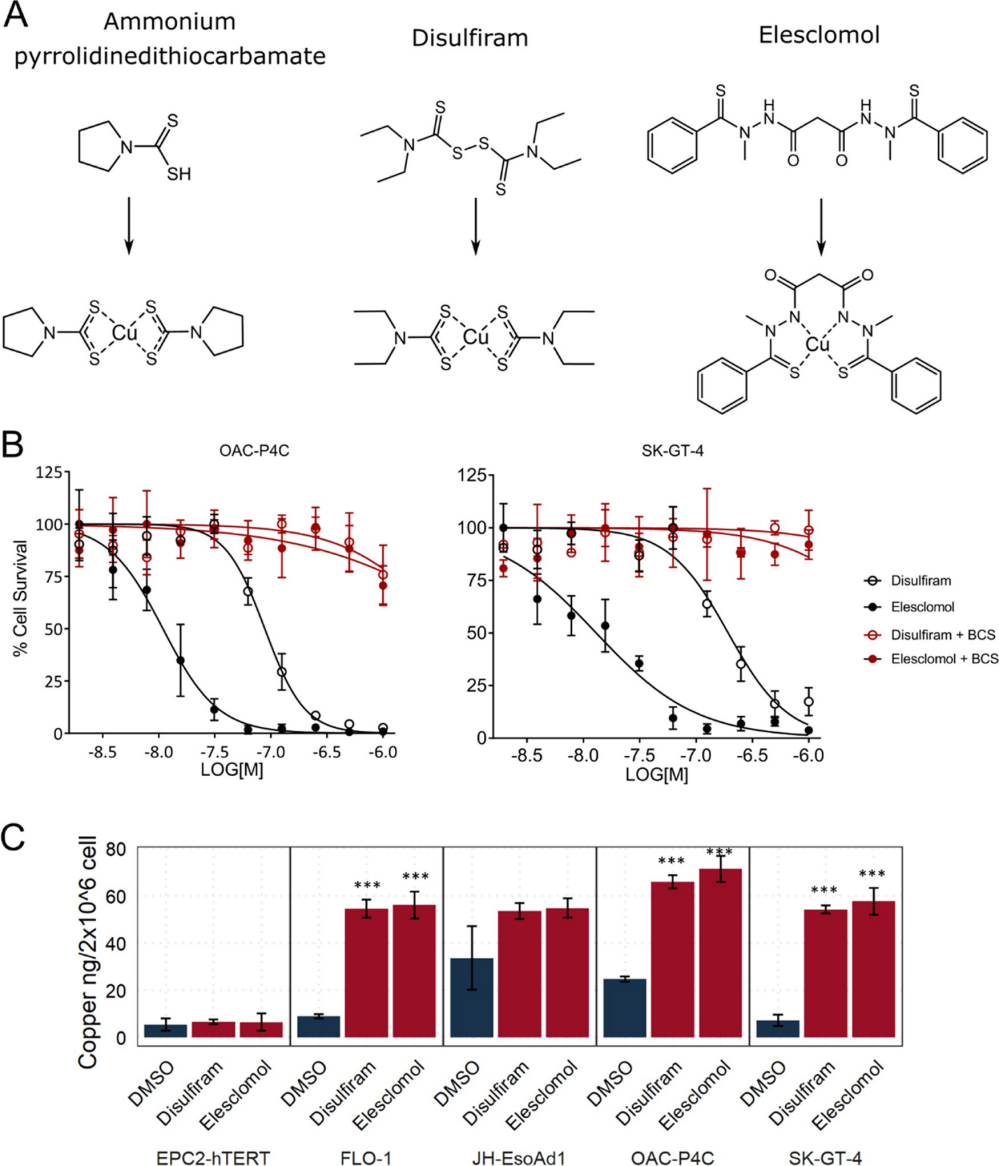
Role of copper in compound activity. (A) Compound structures before and after copper chelation. (B) Dose responses with and without copper chelator bathocuproinedisulfonic acid. (C) Inductively coupled plasma mass spectrometry (ICP-MS) intracellular copper levels. Error bars indicate SE. 1-ANOVA and Tukey’s post-hoc test for significance. Significance indicated for treatments compared to DMSO. ***p < 0.001, *n* = 3.

Disulfiram, elesclomol, and ammonium PDTC are also known to form organometallic complexes with copper^25^ (Figure 4A), and it has been shown previously that elesclomol is capable of shuttling copper into cancer cells.^27^ We therefore wanted to assess the role of copper in the activity of these compounds in EAC selective toxicity. To do this, we assessed whether copper was an essential component of the cell media for cell killing. Preincubation of cells with cell impermeable bathocuproinedisulfonic acid (BCS) to remove free copper from the media led to complete loss of activity for both disulfiram and elesclomol (Figure 4B), confirming free extracellular copper is necessary for the cytotoxic activity of both compounds. In contrast, the iron chelators deferoxamine (data not shown) and ciclopirox olamine (Supporting Figure 1) were unable to rescue cell death, demonstrating specificity for copper.

Using inductively coupled plasma mass spectrometry (ICP-MS) to detect metal ions within cell extracts, we found that both disulfiram and elesclomol treatment led to the accumulation of intracellular copper in EAC cells (Figure 4C), identifying them as copper ionophores in EAC. We also found that other metal ions—Fe, Mg, Mn, and Zn—did not increase with compound treatment (Supporting Figure 7 for Fe levels and data not shown for other metal ions), again demonstrating the specificity for copper in their mechanism of action. Consistent with the lack of cytotoxicity and gene expression changes, ICP-MS showed no accumulation of copper in the tissue-matched control cells after incubation with either disulfiram or elesclomol (Figure 4C). We propose that intracellular copper transport and accumulation is likely the mechanism conferring selectivity over nontransformed cells, though the mechanism leading to the lack of accumulation in nontransformed cells needs to be explored further.

**Figure 5:**
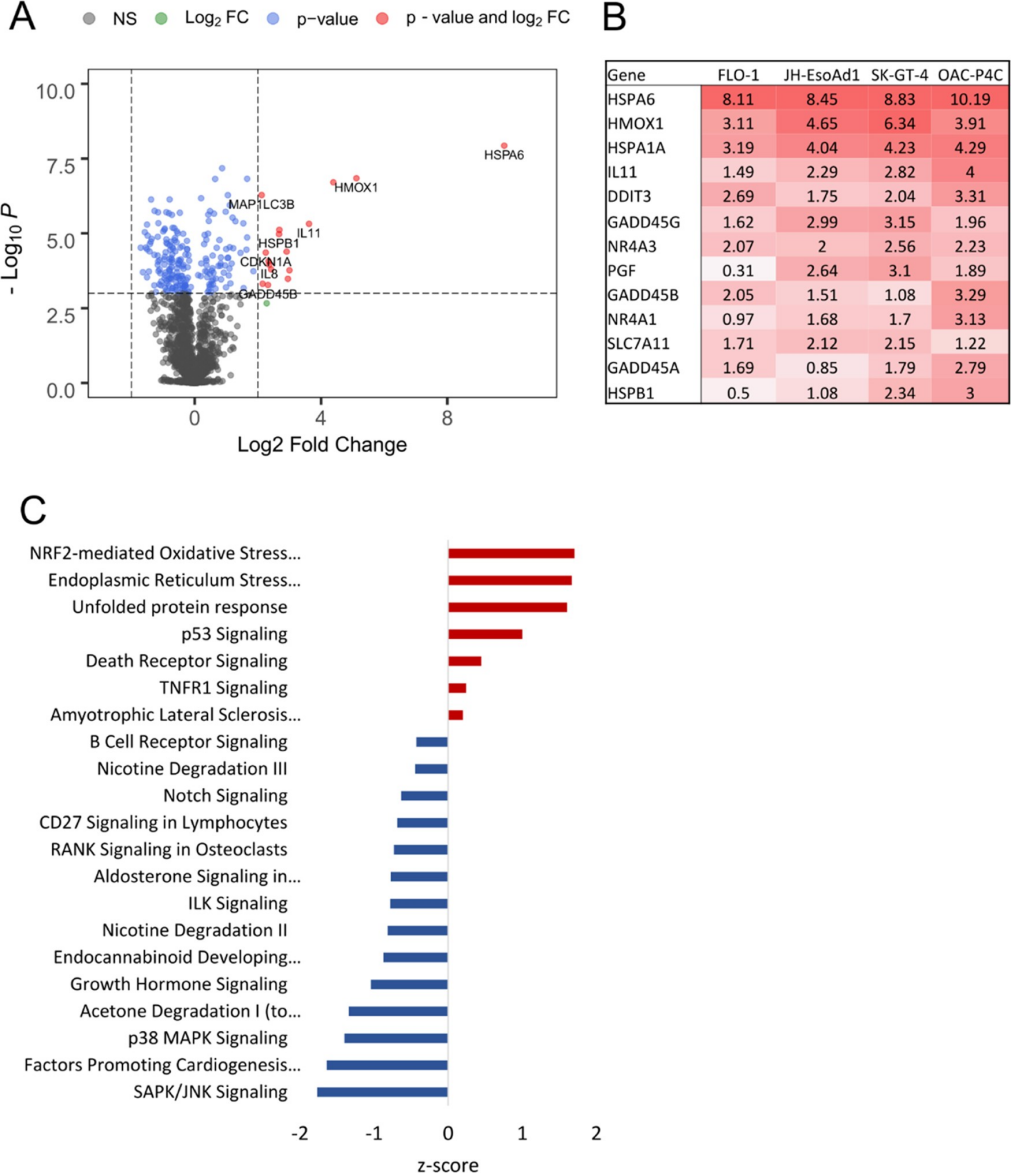
Elesclomol-induced gene expression signature. (A) Differential expression analysis for elesclomol treatment (200 nM for 6 h) vs DSMO in the two most sensitive cell lines OAC-P4C and SK-GT-4. *P*-value cutoff equivalent to adjusted *p*-value 0.05. *n* = 3. (B) Average log 2 fold change for the most significant genes. (C) Top pathway Z-scores (by *p*-value) identified in Ingenuity Pathway Analysis in the sensitive cell lines (OAC-P4C and SK-GT-4).

To further narrow down the mechanisms at play, we took an unbiased approach, focusing on the transcriptomic profile of elesclomol treatment in EAC. NanoString differential expression analysis^35^ revealed strong induction of multiple heat shock, growth arrest and DNA damage genes, and heme oxygenase I (Figure 5A,B). We then used Ingenuity Pathway Analysis to define deregulated pathways in a compound exposure setting to elucidate the mechanism of action. Pathway analysis revealed strong and consistent modulation of SAPK/JNK signaling, NRF2-mediated oxidative stress response, unfolded protein response, endoplasmic reticulum stress, and p53 signaling as the top five altered pathways after elesclomol (Figure 5C) and disulfiram (data not shown) treatment, possibly suggesting a mechanism involving proteotoxic stress.

Given the clear variability in EAC cell line sensitivity to disulfiram and elesclomol, we sought to identify the mechanisms that confer sensitivity through the integration of basal transcriptomic data, as this may further elucidate the mechanism through which they act. Given that TP53 mutations are the most frequent alteration in esophageal cancer^4^ and p53 signaling is one of the top five altered pathways after elesclomol treatment (Figure 5C), we explored potential links between p53 and sensitivity to elesclomol. However, while we saw a very strong correlation between TP53 expression and elesclomol IC_50_, neither mutation nor knockdown of TP53 caused a significant change in elesclomol sensitivity in isogenic cells (Supporting Figure 8).

**Figure 6:**
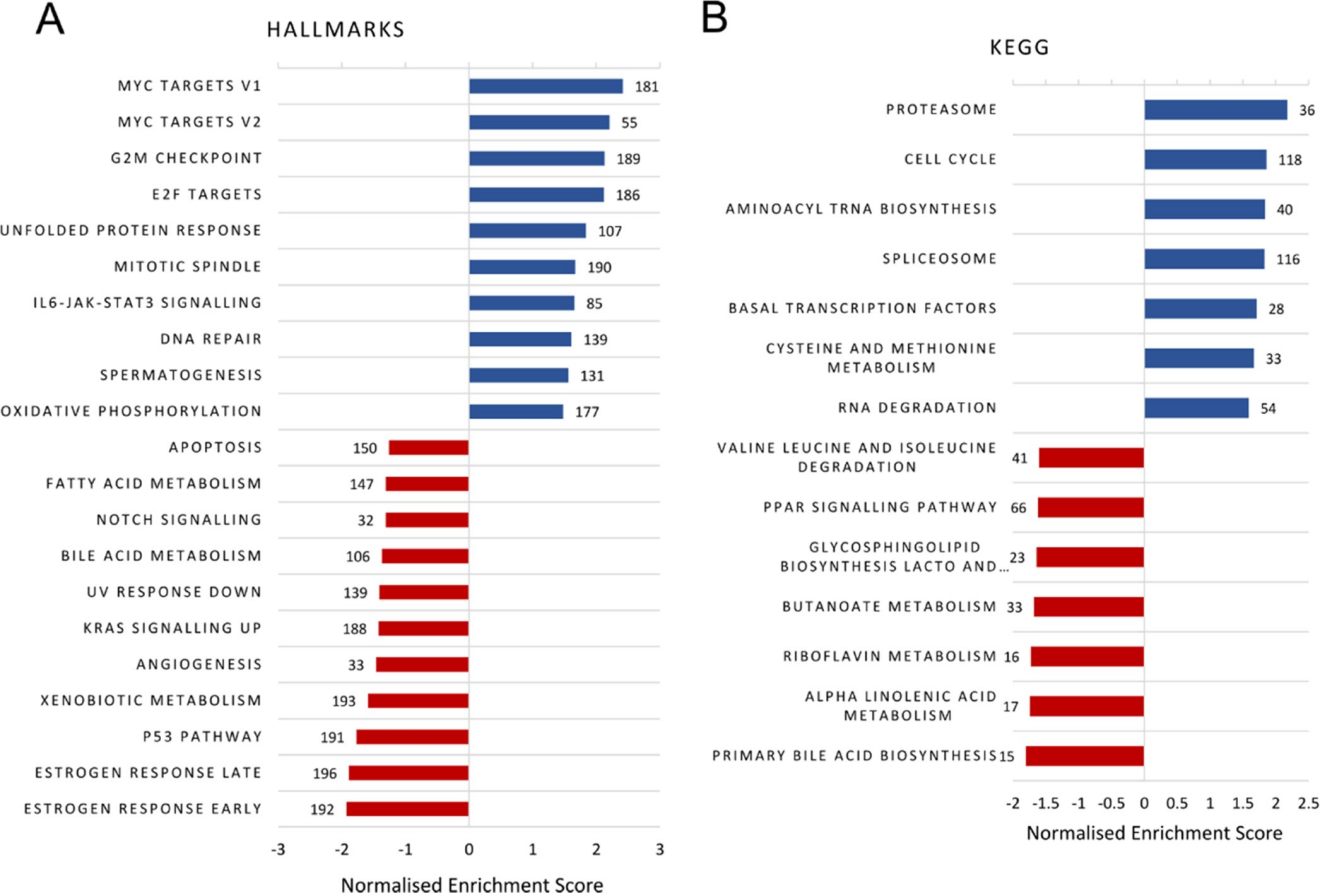
Sensitivity from (A) GSEA Hallmarks analysis using the MSigDB Hallmark collection of 50 gene sets and (B) GSEA KEGG analysis using the canonical pathways KEGG collection of 186 gene sets. Numbers represent gene set size. Pearson was used as a rank metric.

In a more unbiased approach to identify sensitivity mechanisms, we applied gene set enrichment analysis (GSEA) to publicly available gene expression data^36,37^ for seven EAC cell lines to reveal significant associations at the biological pathway level. GSEA of hallmark gene sets revealed 10 that positively correlated and 11 that negatively correlated with IC_50_ (*p* < 0.05) (Figure 6A). MYC targets are the top two gene sets positively associated with elesclomol sensitivity, from which the top differentially expressed genes are ubiquitin enzymes and proteasome subunits (Supporting Table 6). In concordance with this, the unfolded protein response is the fifth gene set identified. Furthermore, GSEA of Kyoto Encyclopedia of Genes and Genomes (KEGG) gene sets revealed 18 that were significantly enriched and positively correlated with elesclomol sensitivity, of which the proteasome was the top gene set (Figure 6B).

We have also carried out differential expression analysis of nine EAC lines profiled with the NanoString nCounter platform and a pan cancer correlation of gene expression using the Genomics of Drug Sensitivity in Cancer data to identify potential sensitivity biomarkers at the gene rather than pathway/network level. Preliminary results suggest a number of potential biomarkers of sensitivity; we highlight methionine sulfoxide reductase B2 (MSRB2), B3 (MSRB3), and glutathione peroxidase 8 (GPX8) as this pathway is consistent across both analyses (Supporting Figure 9). MSRB genes have also been studied in relation to copper overload in yeast and endoplasmic reticulum (ER) stress previously.^38−40^ Follow-up work is required to determine the contribution of these biomarkers to copper-induced cell death in this setting.

Given the identification of upregulated heat shock response and unfolded protein response after compound treatment (known to arise from increased levels of misfolded proteins in the cytosol and endoplasmic reticulum, respectively^41^), ubiquitin and proteasome subunit expression correlating with sensitivity, and the fact that copper is known to bind proteins and induce misfolding and the unfolded protein response,^42−44^ we utilized the morphological data to study potential links to protein misfolding and proteostasis to further elucidate the mechanism by which these compounds work.

**Figure 7:**
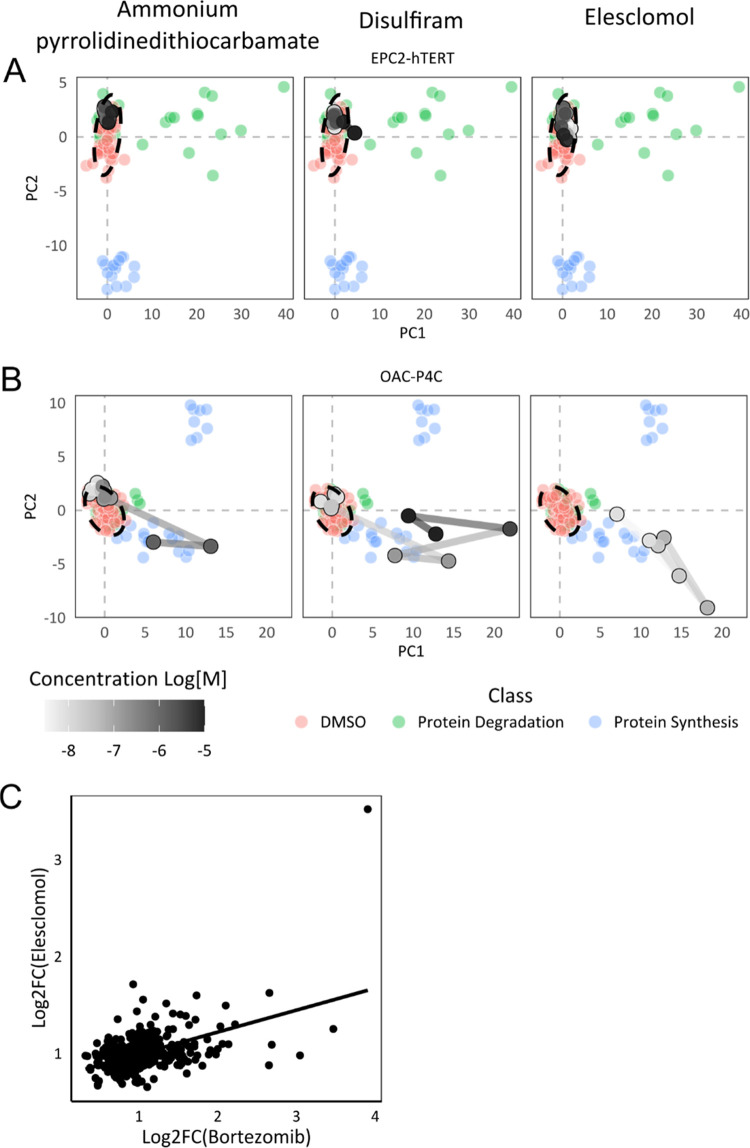
Comparison to compounds affecting proteostasis. First two principal components for (A) tissue-matched, elesclomol-insensitive cell line EPC2-hTERT. (B) Elesclomol-sensitive cell line OAC-P4C. Reference library colored by mechanistic class, dose response colored (grayscale) by concentration. (C) Correlation of bortezomib- and elesclomol-induced gene expression changes. (Data from Tsvetkov et al., 2019).

We quantified the phenotypic responses following treatment with two classes of compounds that affect proteostasis—the proteasome inhibitors MG132 and lactacystin (Figure 7A–C, green points) and the protein synthesis inhibitors cycloheximide and emetine (Figure 7A–C, blue points), and compared them with disulfiram, elesclomol, and ammonium PDTC to determine if they produced a similar cellular phenotype to either compound class, which would suggest a shared mechanism of action. However, phenotypically they do not cluster together, indicating that these copper ionophores do not induce a proteasome, or protein synthesis inhibitor-like phenotype (Figure 7A–C), implying an alternative mechanism. Nevertheless, we noticed a pattern in phenotypic activity across the cell lines when comparing the proteasome inhibitors to the copper ionophores; the proteasome inhibitors caused very limited changes in the copper ionophore-sensitive EAC cell lines (OAC-P4C and SK-GT-4), but caused strong phenotypic changes in the copper ionophore-resistant tissue-matched control cell lines, and the opposite is true for the copper ionophores (Figure 7), suggesting an inverse connection between the two groups of compounds. We hypothesized that this inverse phenotypic activity may indicate that proteasome inhibitor-resistant cells display a unique sensitivity to the copper ionophores, and this has since been proven for elesclomol using proteasome inhibitor-adaptive resistant cells.^45^

To further explore this association, we compared the gene expression signatures induced by bortezomib, a proteasome inhibitor, and elesclomol (data from Tsvetkov et al.).^45^ Unexpectedly, given the lack of similarity in phenotypic response, the two gene expression signatures show a significant correlation (Pearson correlation 0.49, *p* < 0.001) (Figure 7C), although much lower than that between disulfiram and elesclomol. When taken together, we suggest that this similarity in gene expression is because these copper ionophores elicit the same downstream response in cells as the proteasome inhibitors (dysregulation of proteostasis), but critically, we believe this to be via a mechanism that is distinct from proteasome inhibition, given the dissimilarity in morphological signatures and the opposing sensitivity of cells to the two groups of compounds.

Overall, using a panel of cell lines, we applied the Cell Painting assay to identify and cluster selective hit compounds for the treatment of EAC. Then, through the integration of morphological and transcriptomic datasets, we began to deconvolute the mechanism of this cluster of EAC selective compounds, elesclomol, disulfiram, and ammonium PDTC, and identified a unified mechanism of action. We provide evidence toward a mechanism and vulnerability of EAC cells involving an influx of intracellular copper accumulation leading to dysregulation of proteostasis and cancer-specific cell death. This is in line with studies of copper deficiency disorders that demonstrated elesclomol is capable of restoring cuproenzyme activity and alleviating disease symptoms,^46,47^ though we believe this is not via targeted delivery but by simply bypassing the defunct copper transport system in copper disorders and increasing bioavailable copper levels within cells.

Evidence toward a mechanism resulting in dysregulation of proteostasis is provided through the identification of a compound-induced gene expression signature involving pathways known to arise from increased levels of misfolded proteins,^41^ similarity in gene expression signature to known proteasome inhibitors, and identification that proteasome pathway basal gene expression levels predict sensitivity to elesclomol and disulfiram from GSEA analysis. Crucially, given the dissimilarity in morphological signatures and opposing sensitivity of the copper ionophores and known proteasome inhibitors across the EAC panel, we believe that these copper ionophores act via a mechanism that is distinct from the proteasome inhibitors while still inducing the same downstream response—loss of proteostasis. This fits with the finding that disulfiram blocks the cellular machinery involved in misfolded protein response by causing the aggregation of NPL4^21^ but does not affect the CT-like, C-like, or T-like activity of 20S proteasome, and recent findings that elesclomol causes the aggregation of lipoylated proteins.^30^ In this current work, we bring these separate observations for disulfiram and elesclomol together by suggesting that protein aggregation is a nonspecific and global response to copper ionophores and is not due to the aggregation of any one specific protein target. Further, our conclusions fit with recent data demonstrating that copper ions interact with proteins to impair folding and promote protein aggregation.^30,44^

While not the focus of our work presented here, we do acknowledge that a role for metabolism and mitochondrial respiration in elesclomol sensitivity has been suggested in previous work.^30,45,48^ Since links between the proteasome and cellular metabolism have been established in other areas of research,^49,50^ we do not believe our current findings relating to elesclomol and proteostasis are mutually exclusive from those relating to metabolism. In our work, we do note the identification of multiple metabolic pathways, including oxidative phosphorylation, fatty acid metabolism, glycosphingolipid biosynthesis, and bile acid metabolism, in our copper ionophore sensitivity GSEA (Figure 6). Further, we have performed oxygen consumption rate (OCR) experiments, which agree with recent findings that at low but relevant doses of elesclomol, there is no effect on basal or ATP-linked mitochondrial respiration; however, there is a significant reduction in spare respiratory capacity (Supporting Figure 10). Future studies to fully understand the role of metabolism and mitochondrial respiration and its links to proteostasis in the specific context of copper ionophores may shed further light on the mechanisms at play.

In conclusion, this work provides a model framework to identify and deconvolute new therapeutic targets and classes of small molecules utilizing a multipronged, multitechnology, holistic approach. We believe the holistic and target-agnostic approach we have employed in this study across panels of cell lines embraces the complexity of heterogeneous diseases and can be applied early on in the drug discovery pipeline to have a beneficial impact upon drug discovery success rates as a whole in such disease areas of unmet need.

## Materials and Methods

### Cell Culture

EAC lines were grown in RPMI-1640 (Life Technologies; #11875101) with 10% fetal bovine serum (FBS) (Life Technologies; #16140071) and 2 mM l-glutamine (Life Technologies; #A2916801). CP-A and EPC2-hTERT cells were grown in KSFM (Life Technologies; #17005075) with 5 g L^–1^ human recombinant epidermal growth factor and 50 mg L^–1^ bovine pituitary extract (Supporting Table 7 for cell line details).

Primary organoid cultures were derived from normal gastric and EAC cases included in esophageal cancer clinical and molecular stratification (OCCAMS)/international cancer genome consortium (ICGC) sequencing study. Detailed organoid culture and derivation method have been previously described in detail.^29^ Cells were seeded in a complete medium and then treated with elesclomol in 9-point half-log serial dilution for 6 days (maximal concentration 10 μM). Treatments were performed in technical duplicates and at least two biological replicates. Cell viability was assessed using CellTiter-Glo (Promega).

### Compound Screening

Cells were seeded at 800–1500 cells per well in 50 μL into 384-well microplates (Greiner, #781091) for 24 h. Compounds were then added as 8-point semi-log dose responses from 10 μM before being incubated for a further 48 h.

For morphological and nuclei count readouts, the Cell Painting protocol was applied, which uses multiplexed fluorescent dyes to visualize cellular and subcellular organelle and cytoskeletal morphology.^12,13^ Cells were fixed in 4% formaldehyde before permeabilization in 0.1% Triton-X100 (v/v). Finally, the staining solution was added in 1% bovine serum albumin in PBS (w/v) and incubated for 30 min before being washed out. (Supporting Table 8). Four fields of view were captured per well using a 20× objective and five filters (Supporting Table 8).

For the expanded panel of EAC cell lines, data CellTitre-Glo (promega) was added according to the manufacturer’s instructions and incubated for 10 min at room temperature before being read. For caspase-dependent apoptosis assays, we monitored caspase activity at sequential time points following compound treatments using the Incucyte imaging instrument and caspase-3/7 biosensor according to the manufacturer’s instructions (Sartorius).

### Image Analysis

CellProfiler v3.1.5 software was used to extract features from the images. The cell-level data was aggregated to the image level by taking the median for each measured feature per image. Low-quality images and image artifacts were then identified and removed using image quality metrics extracted by CellProfiler. Images with 20 cells or less were also removed from the dataset. For the remaining images, features were normalized on a plate-by-plate basis by dividing each feature by the median DMSO response for that feature. Features with NA values were removed, as were features with zero or near-zero variance, using the “findCorrelation” and “nearZero” functions in the R package Caret. All remaining features were scaled and centered globally by dividing by the standard deviation of each feature and subtracting the feature mean, respectively. The pairwise correlations were calculated for all remaining features, and highly correlated features (>0.90) were removed. Finally, the image data was aggregated to the well (compound) level, and this was used in the analysis.

All analysis was conducted in R http://www.r-project.org using software packages available via CRAN http://cran.r-project.org and Bioconductor http://www.bioconductor.org.

Copper chelator bathocuproinedisulfonic acid (BCS). The dose responses were carried out as stated for the validation dose responses, except that disulfiram and elesclomol dose responses were carried out in the presence or absence of 200 μM BCS preincubation for 15 min.

### Transcriptomic Analyses

Cells were seeded at 8 × 10^4^ cells in 6-well plates for 24 h. Media was replaced with fresh media containing compound treatments (DMSO (0.1%), disulfiram (600 nM), or elesclomol (200 nM)) before further incubation for 6 h. Media was then removed, and plates were washed twice with ice-cold PBS before being snap-frozen at −80. Cells were scraped and lysed using QIAshredders (#79654, QIAGEN) and Qiagen RNeasy Mini kit (#74104, QIAGEN) (with β-mercaptoethanol) according to the manufacturer’s instructions and included a DNase digestion step (#79254, QIAGEN). Briefly, 100 ng of each sample was loaded into the NanoString nCounter Analysis System with the Human PanCancer Pathways and Metabolic Pathways panels. Raw counts were normalized to the internal positive controls and housekeeping genes using the nSolver 4.0 software. nSolver 4.0 software and the NanoStringDiff algorithm^35^ were used for differential expression analysis. *P*-values were adjusted using the Benjamini–Yekutieli approach.^54^ Treatment-induced analysis was carried out between the control (DMSO) and treatment (200 nM elesclomol) samples for the two most sensitive cell lines (OAC-P4C and SK-GT-4) pooled together. *N* = 3. The difference in cell lines was taken into account as a confounder in the analysis.

### Intracellular Copper Quantification

Typically, 5 × 10^6^ cells were seeded in a T175 flask and incubated for 24 h. Media was replaced with compound treatments (DMSO (0.1%), disulfiram (600 nM), or elesclomol (200 nM)) before further incubation for 6 h. Cells were washed in PBS, trypsinized, and counted. For each sample, 2 × 10^6^ cells were pelleted and frozen at −80 °C. For analysis, samples were thawed, and concentrated nitric acid was added (100 μL) and mixed. Samples were then vortexed and sonicated and left overnight at room temperature. Samples were made up to 1 mL using water and then further diluted 5-fold prior to analysis of copper content by inductively coupled plasma mass spectrometry (ICP-MS).

### Gene Set Enrichment Analysis

Enrichment analysis was conducted using GSEA,^51^ on the genomics of drug sensitivity in cancer Robust Multichip Average (RMA) processed gene expression data (GDSC1000, see below for download link) for the cell lines FlO-1, OE19, OE33, OAC-M5.1, OAC-P4C, SK-GT-4, ESO26, ESO51, and our own IC_50_ sensitivity data for elesclomol, using the H and C2 gene sets from the molecular signatures database (MSigDB; see below for details). Default settings were used, except that Pearson was used for gene ranking.

H-Hallmark gene set collection includes 50 gene sets.^52^

C2-Canonical pathways KEGG collection includes 186 gene sets^53^

GDSC1000 downloads:

The Robust Multichip Average (RMA)-processed dataset is available at http://www.cancerrxgene.org/gdsc1000//Data/preprocessed/Cell_line_RMA_proc_basalExp.txt.zip.

## Supporting Information Available

Validation compound annotations and AUC values (XLSX)
Primary screening and validation hits, reference library compound annotations, compound of interest IC_50_ values, cell line panel annotations, and Cell Painting reagents (Tables S1–S8); nontransformed cell line compound-induced phenotypes and genotypes, cell painting images, and additional negative results (Figures S1–S7) (PDF)
