# Supplementary material for: Multiparametric High-Content Cell Painting Identifies Copper Ionophores as Selective Modulators of Esophageal Cancer Phenotypes
Source: ACS Chem Biol. 2022 Jun 13;17(7):1876–89. doi: 10.1021/acschembio.2c00301 (PMC9295120; doi:10.1021/acschembio.2c00301)
Supplement: Supplementary file 2 — cb2c00301_si_002.pdf [file cb2c00301_si_002.pdf]

Supporting Information

Title: Multiparametric High Content Cell Painting Identifies Copper Ionophores as Selective Modulators of Esophageal Cancer Phenotypes

Authors:

Rebecca E. Hughes[1], Richard J. R. Elliott[1], Xiaodun Li[2], Alison F. Munro[1], Ashraff Makda[1], Roderick N. Carter[3,4], Nicholas M. Morton[4], Kenji Fujihara[5,6], Nicholas J. Clemons[5,6], Rebecca Fitzgerald[2], J. Robert O'Neill[7], Ted Hupp[1], Neil O. Carragher*[1]

Affiliations:

[1] Cancer Research UK Edinburgh Centre, Institute of Genetics & Cancer, The University of Edinburgh, Western General Hospital, Edinburgh, EH4 2XR, UK

[2] MRC Cancer Unit, Hutchison-MRC Research Centre, University of Cambridge, Cambridge, CB2 0XZ, UK

[3] Centre for Clinical Brain Sciences, Chancellors Building, University of Edinburgh, Edinburgh, EH16 4SB, UK.

[4] Centre for Cardiovascular Science, The Queen's Medical Research Institute, Edinburgh BioQuarter, Edinburgh, EH16 4TJ, UK.

[5] Gastrointestinal Cancer Program, Cancer Research Division, Peter MacCallum Cancer Centre, Melbourne 3000, Victoria, Australia.

[6] Sir Peter MacCallum Department of Oncology, The University of Melbourne, Parkville 3010, Victoria, Australia.

[7] Cambridge Oesophagogastric Centre, Cambridge University Hospitals Foundation Trust, Cambridge, CB2 2QQ, UK.

Corresponding author

Neil Carragher: N.Carragher@ed.ac.uk

Supplementary Table 1: Primary screen compound hits. *Not available for rescreening

	Phenotypic hits	cell survival hits
1	(S)-(+)-Camptothecin	
2	5-Fluorouracil	Adrucil(Fluorouracil)
3	Alcuronium chloride	
4	Aminopterin	
5		Ammonium pyrrolidinedithiocarbamate
6	Amonafide	
7	Amsacrine hydrochloride	
8	Ancitabine hydrochloride	
9	Ascorbic acid	Ascorbic acid
10		Aurora A Inhibitor I
11	Azapropazone	
12	AZD7762	
13	Bacampicillin hydrochloride*	Bacampicillin hydrochloride*
14	Benzylpenicillin sodium	Benzydamine hydrochloride
15	Betahistine mesylate	
16	BI 2536	
17	BIX-01294·3HCl	
18	Calcimycin	
19	Cantharidic Acid	
20	Cantharidin	
21	Carmofur	
22	CCT137690	
23	Ceforanide	
24	CID 11210285 hydrochloride	
25		Clindamycin hydrochloride
26	Clofarabine	
27		Clorgyline hydrochloride
28	Colchicine	
29	Cytosine-1-beta-D-arabinofuranoside hydrochloride	
30	Deptropine citrate	Deptropine citrate
31	Dipivefrin hydrochloride	Dipivefrin hydrochloride
32		Disulfiram
33		Elesclomol

34	Ellence	
35		Etifenin
36	Etoposide	
37	Floxuridine	Floxuridine
38	Fluoxetine hydrochloride	Fluoxetine hydrochloride
39	Gemcitabine hydrochloride	
40	Ibuprofen	
41	JNJ-26481585	
42	Letrozole	
43	Methotrexate	Methotrexate
44	Mitoxantrone	
45	Mizolastine	
46	MK-2206	MK-2206
47	MLN8237	
48	Molsidomine	
49		Nicergoline
50	NSC-3852	NSC-3852
51	Obatoclax Mesylate	
52		Oxytetracycline dihydrate
53	Paclitaxel	
54	Pemetrexed disodium (LY-231514)	Pemetrexed disodium (LY-231514)
55	Pirenperone	
56	PKC-412	
57	PMEG hydrate*	
58	Podophyllotoxin	
59		Propylthiouracil
60	Raltitrexed (Tomudex)	Raltitrexed(Tomudex)
61	SB743921 hydrochloride	
62	SB939	
63	SNS-032(BMS-387032)	
64	SNS-314 Mesylate	
65	Suprafenacine	
66		Terbutaline hemisulfate
67	Thiocolchicine	
68	Thiorphan	Thiorphan
69	Topotecan hydrochloride hydrate	

70	Trichostatin A	Trichostatin A
71	Vincristine sulfate	
72	Vinorelbine (Navelbine)	
73	VX-680	
74		YM155

Supplementary Table 2. Dose response validation compounds. (Red- not selective)

	validation compounds	Phenotypic Validation	Cell survival Validation
1	(S)-(+)-Camptothecin	✓	✓
2	5-Fluorouracil	✓	✓
3	alcuronium chloride		
4	Aminopterin		✓
5	Ammonium pyrrolidinedithiocarbamate	✓	✓
6	Amonafide	✓	✓
7	Amsacrine hydrochloride	✓	✓
8	Ancitabine hydrochloride	✓	✓
9	Ascorbic acid		
10	Aurora A Inhibitor I	✓	✓
11	Azapropazone		
12	AZD7762	✓	✓
13	Benzydamine hydrochloride	✓	
14	Benzylpenicillin sodium		
15	Betahistine mesylate		
16	BI 2536	✓	✓
17	BIX 01294 trihydrochloride hydrate	✓	
18	Calcimycin	✓	✓
19	Cantharidic Acid	✓	
20	Cantharidin	✓	✓
21	Carmofur	✓	✓
22	CCT137690	✓	✓
23	Ceforanide		
24	CID 11210285 hydrochloride	✓	✓
25	Clindamycin hydrochloride		
26	Clofarabine	✓	✓

27	Clorgyline hydrochloride		
28	Colchicine	✓	✓
29	Cytosine-1-beta-D-arabinofuranoside hydrochloride	✓	✓
30	deptropine citrate		
31	Dipivefrin hydrochloride		
32	Disulfiram	✓	✓
33	Elesclomol	✓	✓
34	Ellence	✓	✓
35	Etifenin		
36	Etoposide	✓	✓
37	Floxuridine	✓	✓
38	Fluoxetine hydrochloride	✓	
39	Gemcitabine hydrochloride	✓	✓
40	Ibuprofen (racemic)		
41	JNJ-26481585	✓	✓
42	Letrozole		
43	Methotrexate	✓	✓
44	Mitoxantrone	✓	✓
45	Mizolastine		
46	MK-2206	✓	✓
47	MLN8237	✓	✓
48	Molsidomine		
49	Nicergoline		
50	NSC-3852	✓	✓
51	Obatoclax Mesylate	✓	✓
52	Oxytetracycline dihydrate		
53	Paclitaxel	✓	✓
54	Pemetrexed disodium (LY-231514)	✓	✓
55	pirenperone		
56	PKC-412		✓
57	Podophyllotoxin		✓
58	Propylthiouracil		
69	Raltitrexed(Tomudex)	✓	✓
60	SB743921 hydrochloride	✓	✓
61	SNS-032(BMS-387032)	✓	✓

62	SNS-314 (mesylate)	✓	✓
63	Suprafenacine	✓	✓
64	Terbutaline hemisulfate		
65	Thiocolchicine	✓	✓
66	Thiorphan		
67	Topotecan hydrochloride hydrate	✓	✓
68	Trichostatin A	✓	✓
69	Vincristine sulfate	✓	✓
70	Vinorelbine(Navelbine)	✓	✓
71	VX-680	✓	✓
72	YM155	✓	✓

Supplementary Table 3: Mechanism of action reference compounds. Compound name, mechanism of action, sub-class and supplier and catalogue number.

Compound Name	Mechanism of Action	Sub-Class	Supplier; Catalogue number
Cytochalasin B	Actin disrupting	Actin disruptor	Sigma; C8273
Cytochalasin D	Actin disrupting	Actin disruptor	Sigma; C6762
Latrunculin	Actin disrupting	Actin stabiliser	Sigma; L5288
Camptothecin	DNA damaging	Topoisomerase-1 inhibitor	Selleckchem; S1288
SN38	DNA damaging	Topoisomerase-1 inhibitor	Selleckchem; S4908
Dasatinib	Kinase inhibitor	Src- EMT	Selleckchem; S1021
Saracatinib	Kinase inhibitor	Src-EMT	Selleckchem; S1006
Epothilone B	Microtubule disrupting	Microtubule stabiliser	Selleckchem; S1364
Paclitaxel	Microtubule disrupting	Microtubule stabiliser	Sigma; T7402
Colchicine	Microtubule disrupting	Microtubule destabiliser	Sigma; C9754
Nocodazole	Microtubule disrupting	Microtubule destabiliser	Sigma; M1404
Monastrol	Microtubule disrupting	Eg5 kinesin inhibitor	Sigma; M8515
ARQ621	Microtubule disrupting	Eg5 kinesin inhibitor	Selleckchem; S7355
Barasertib	Microtubule disrupting	Aurora kinase B inhibitor	Selleckchem; S1147
ZM447439	Microtubule disrupting	Aurora kinase B inhibitor	Selleckchem; 1103
MG132	Protein degradation	Proteasome	Selleckchem; S2619
Lactacystin	Protein degradation	Proteasome	Tocris; 2267
ALLN	Protein degradation	Cysteine/calpain	Sigma; A6165
ALLM	Protein degradation	Cysteine/calpain	Sigma; A6060
Cycloheximide	Protein synthesis	Protein synthesis	Sigma; 01810

Emetine	Protein synthesis	Protein synthesis	Sigma; E2375
Lovastatin	Statin	Statin	Sigma; PHR1285
Simvastatin	Statin	Statin	Sigma; PHR1438
SAHA	HDAC inhibitor	HDAC inhibitor	Sigma; SML0061
Panobinostat	HDAC inhibitor	HDAC inhibitor	Selleckchem; S1030
Trichostatin A	HDAC inhibitor	HDAC inhibitor	Selleckchem; S1045
Romidepsin	HDAC inhibitor	HDAC inhibitor	Selleckchem; S3020
Entinostat	HDAC inhibitor	HDAC inhibitor	Selleckchem; S1053
Quisinostat	HDAC inhibitor	HDAC inhibitor	Selleckchem; S1096
Ricolinostat	HDAC inhibitor	HDAC inhibitor	Selleckchem; S8001
Tubastatin A	HDAC inhibitor	HDAC inhibitor	Selleckchem; S8049
Droxinostat	HDAC inhibitor	HDAC inhibitor	Selleckchem; S1422
PCI34051	HDAC inhibitor	HDAC inhibitor	Selleckchem; S2021
TMP195	HDAC inhibitor	HDAC inhibitor	Selleckchem; S8502
LMK235	HDAC inhibitor	HDAC inhibitor	Selleckchem; S7569
CUDC-907	HDAC inhibitor	HDAC inhibitor	Selleckchem; S2759
Belinostat	HDAC inhibitor	HDAC inhibitor	Selleckchem; S1085

Supplementary Table 4: Compound normalised Area Under the Curve (AUC) values across panel of cell lines.

	Ammmonium pyrrolidinediothiocarbamate			Disulfiram			Elesclomol		
Cell line	Normalised AUC	95% Confidence Interval		Normalised AUC	95% Confidence Interval		Normalised AUC	95% Confidence Interval	
		Lower limit	Upper Limit		Lower limit	Upper Limit		Lower limit	Upper Limit
CP-A	1.026	0.998	1.055	0.978	0.933	1.025	1.062	1.025	1.100
EPC2-hTERT	0.995	0.945	1.045	0.958	0.913	1.003	0.864	0.828	0.900
JH-EsoAd1	0.915	0.858	0.973	0.897	0.850	0.945	0.738	0.685	0.793
FLO-1	0.963	0.913	1.010	0.826	0.790	0.863	0.833	0.783	0.883
MFD-1	0.872	0.800	0.943	0.629	0.563	0.693	0.512	0.468	0.558
OE33	0.882	0.838	0.928	0.654	0.620	0.688	0.451	0.383	0.520
SK-GT-4	0.863	0.825	0.903	0.585	0.545	0.625	0.353	0.315	0.393
OAC-P4C	0.706	0.668	0.743	0.474	0.445	0.503	0.137	0.108	0.168

Supplementary Table 5: Patient derived organoids. Elesclomol normalised Area Under the Curve (AUC) values.

Cell line	Normalised AUC	95% Confidence Interval	
		Lower limit	Upper Limit
NG088	0.83	0.78	0.87
CAM277	0.48	0.46	0.50
CAM401	0.56	0.52	0.61
CAM408	0.42	0.40	0.45
CAM479	0.50	0.46	0.55
CAM486	0.35	0.30	0.41

Supplementary Table 6: Rank order for core enriched genes in the MYC 1 Hallmark Geneset.

Number	Gene Symbol	Gene Name	Rank
1	UBA2	ubiquitin like modifier activating enzyme 2	87
2	PSMD8	proteasome 26S subunit, non-ATPase 8	212
3	SRM	spermidine synthase	215
4	LSM2	LSM2 homolog, U6 small nuclear RNA and mRNA degradation associated	267
5	RACK1	receptor for activated C kinase 1	287
6	PSMC4	proteasome 26S subunit, ATPase 4	375
7	PWP1	PWP1 homolog, endonuclein	376
8	UBE2E1	ubiquitin conjugating enzyme E2 E1	479
9	SF3A1	splicing factor 3a subunit 1	507
10	SF3B3	splicing factor 3b subunit 3	575
11	PABPC4	poly(A) binding protein cytoplasmic 4	725
12	PHB2	prohibitin 2	751
13	SLC25A3	solute carrier family 25 member 3	860
14	UBE2L3	ubiquitin conjugating enzyme E2 L3	875
15	USP1	ubiquitin specific peptidase 1	887
16	VDAC3	voltage dependent anion channel 3	954
17	SNRPA	small nuclear ribonucleoprotein polypeptide A	1055
18	XPOT	exportin for tRNA	1133
19	MRPL9	mitochondrial ribosomal protein L9	1155
20	SMARCC1	SWI/SNF related, matrix associated, actin dependent regulator of chromatin subfamily c member 1	1161
21	NAP1L1	nucleosome assembly protein 1 like 1	1171
22	RANBP1	RAN binding protein 1	1353
23	G3BP1	G3BP stress granule assembly factor 1	1366
24	PTGES3	prostaglandin E synthase 3	1381
25	HNRNPC	heterogeneous nuclear ribonucleoprotein C	1384
26	MRPS18B	mitochondrial ribosomal protein S18B	1403
27	CCT2	chaperonin containing TCP1 subunit 2	1429
28	SRPK1	SRSF protein kinase 1	1490

29	NOLC1	nucleolar and coiled-body phosphoprotein 1	1512
30	RAN	RAN, member RAS oncogene family	1551
31	HNRNPR	heterogeneous nuclear ribonucleoprotein R	1565
32	MCM5	minichromosome maintenance complex component 5	1610
33	CDK4	cyclin dependent kinase 4	1636
34	LSM7	LSM7 homolog, U6 small nuclear RNA and mRNA degradation associated	1652
35	VBP1	VHL binding protein 1	1661
36	GNL3	G protein nucleolar 3	1724
37	CDC45	cell division cycle 45	1769
38	CSTF2	cleavage stimulation factor subunit 2	1802
39	CDC20	cell division cycle 20	1807
40	RSL1D1	ribosomal L1 domain containing 1	1820
41	KARS1	lysyl-tRNA synthetase 1	1885
42	U2AF1	U2 small nuclear RNA auxiliary factor 1	1925
43	PSMA4	proteasome 20S subunit alpha 4	1931
44	EIF3B	eukaryotic translation initiation factor 3 subunit B	1978
45	CAD	carbamoyl-phosphate synthetase 2, aspartate transcarbamylase, and dihydroorotase	2065
46	YWHAE	tyrosine 3-monooxygenase/tryptophan 5-monooxygenase activation protein epsilon	2083
47	FBL	fibrillarin	2138
48	TRA2B	transformer 2 beta homolog	2140
49	XPO1	exportin 1	2151
50	PPM1G	protein phosphatase, Mg2+/Mn2+ dependent 1G	2423
51	ETF1	eukaryotic translation termination factor 1	2500
52	TXNL4A	thioredoxin like 4A	2578
53	EIF4E	eukaryotic translation initiation factor 4E	2646
54	POLD2	DNA polymerase delta 2, accessory subunit	2664
55	EIF3J	eukaryotic translation initiation factor 3 subunit J	2667
56	TUFM	Tu translation elongation factor, mitochondrial	2700
57	IMPDH2	inosine monophosphate dehydrogenase 2	2734
58	PSMB2	proteasome 20S subunit beta 2	2808

59	PSMD14	proteasome 26S subunit, non-ATPase 14	2847
60	EIF1AX	eukaryotic translation initiation factor 1A X-linked	2855
61	NPM1	nucleophosmin 1	2877
62	C1QBP	complement C1q binding protein	2934
63	BUB3	BUB3 mitotic checkpoint protein	3008
64	POLE3	DNA polymerase epsilon 3, accessory subunit	3093
65	RRP9	ribosomal RNA processing 9, U3 small nucleolar RNA binding protein	3205
66	NHP2	NHP2 ribonucleoprotein	3209
67	LDHA	lactate dehydrogenase A	3235
68	NOP16	NOP16 nucleolar protein	3266
69	SNRPD3	small nuclear ribonucleoprotein D3 polypeptide	3334
70	CCT4	chaperonin containing TCP1 subunit 4	3422
71	DEK	DEK proto-oncogene	3531
72	PA2G4	proliferation-associated 2G4	3591
73	CCT7	chaperonin containing TCP1 subunit 7	3647
74	NDUFAB1	NADH:ubiquinone oxidoreductase subunit AB1	3668
75	TCP1	t-complex 1	3695
76	PSMD7	proteasome 26S subunit, non-ATPase 7	3716
77	GLO1	glyoxalase I	3749
78	CTPS1	CTP synthase 1	3838
79	PSMD1	proteasome 26S subunit, non-ATPase 1	3883
80	SSB	small RNA binding exonuclease protection factor La	3961
81	PCBP1	poly(rC) binding protein 1	4034
82	RPL14	ribosomal protein L14	4035
83	HPRT1	hypoxanthine phosphoribosyltransferase 1	4109
84	EPRS1	glutamyl-prolyl-tRNA synthetase 1	4111
85	TARDBP	TAR DNA binding protein	4122
86	EIF3D	eukaryotic translation initiation factor 3 subunit D	4147
87	MRPL23	mitochondrial ribosomal protein L23	4192
88	ODC1	ornithine decarboxylase 1	4203
89	RFC4	replication factor C subunit 4	4218

90	AIMP2	aminoacyl tRNA synthetase complex interacting multifunctional protein 2	4249
91	TYMS	thymidylate synthetase	4371
92	CYC1	cytochrome c1	4420
93	PRPS2	phosphoribosyl pyrophosphate synthetase 2	4436
94	APEX1	apurinic/apyrimidinic endodeoxyribonuclease 1	4457

Supplementary Table 7: Patient and tumour origin and characteristics of oesophageal adenocarcinoma cell lines. GOJ, gastroesohageal junction

Cell Line	Gender	Age	Ethnicity	Barrett's	Location	Grade	Stage	Sequencing study
SK-GT-4	Male	89	White	Yes	Distal 1/3	Well	pT2N1Mx	[1]
FLO-1	Male	68	White	No	Distal 1/3	Poor	pT2N1M0	[1,2]
OE33	Female	73	White	Yes	Distal 1/3	Poor	pT3N0M0	[1,2]
OAC-P4C	Male	55	White	No	GOJ	Moderate	pT3N1M1	[1]
JH-EsoAd1	Male	66	White	Yes	Distal 1/3	Moderate	pT3N0M0	[1]
MFD-1	Male	55	White	No	Distal 1/3	Moderate	pT4N3M0	[2]

1. Contino G, Eldridge MD, Secrier M, et al. Whole-genome sequencing of nine esophageal adenocarcinoma cell lines. F1000Research 2016;5:1336.

2. Garcia E, Hayden A, Birts C, et al. Authentication and characterisation of a new oesophageal adenocarcinoma cell line: MFD-1. Sci Rep 2016;6:32417.

Supplementary Table 8: Cell Painting reagents. Concentrations, excitation/emission wavelengths of the filters used for imaging, and suppliers. ex: excitation, em: emission

Stain	Structure	Wavelength (ex/em [nm])	Channel	Concentration	Cat No; Supplier
Hoescht 33342	Nuclei	387/447	DAPI	4 µg/mL	#H1399; Mol. Probes
SYTO 14	Nucleoli	531/593	CY3	3 µM	#S7576; Invitrogen
Phalloidin 594	F-actin	562/624	TxRED	0.14X	#ab176757; Abcam
Wheat germ agglutinin Alexa Fluor 594	Golgi and Plasma Membrane	562/624	TxRED	1 µg/mL	#W11262; Invitrogen
Concanavalin A Alexa Fluor 488	Endoplas-mic Reticulum	462/520	FITC	20 µg/mL	#C11252; Invitrogen
MitoTracker DeepRed	Mitochond-ria	628/692	CY5	600 nM	#M22426; Invitrogen

Supplementary Figures.

A.

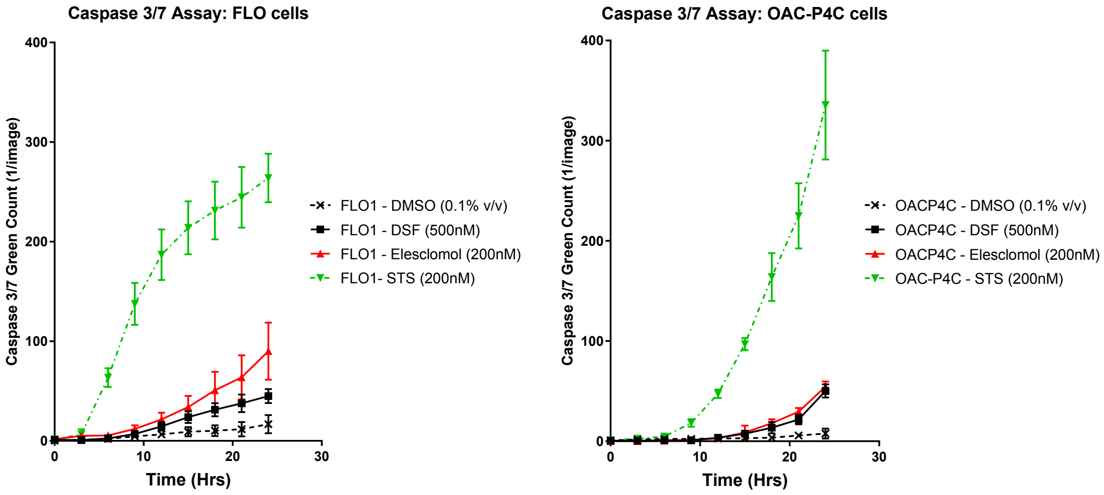

B.

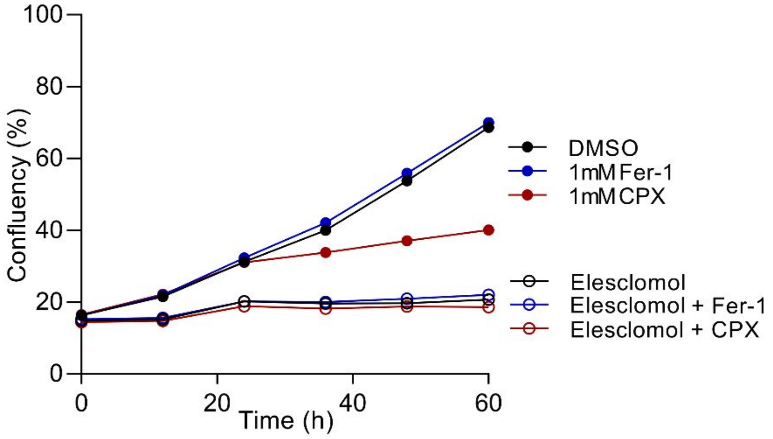

Supplementary Figure 1: Caspase and ferroptosis. A) Caspase 3/7 cell count for resistant line FLO-1 and sensitive line OAC-P4C after treatment with elesclomol and disulfiram (DSF), compared with known apoptosis inducer staurosporin (STS). **B)** OAC-P4C cell confluency after treatment with elesclomol (10 µM) in the presence or absence of the iron chelator ciclopirox olamine (CPX), and ferroptosis inhibitor Ferrostatin-1 (Fer-1).

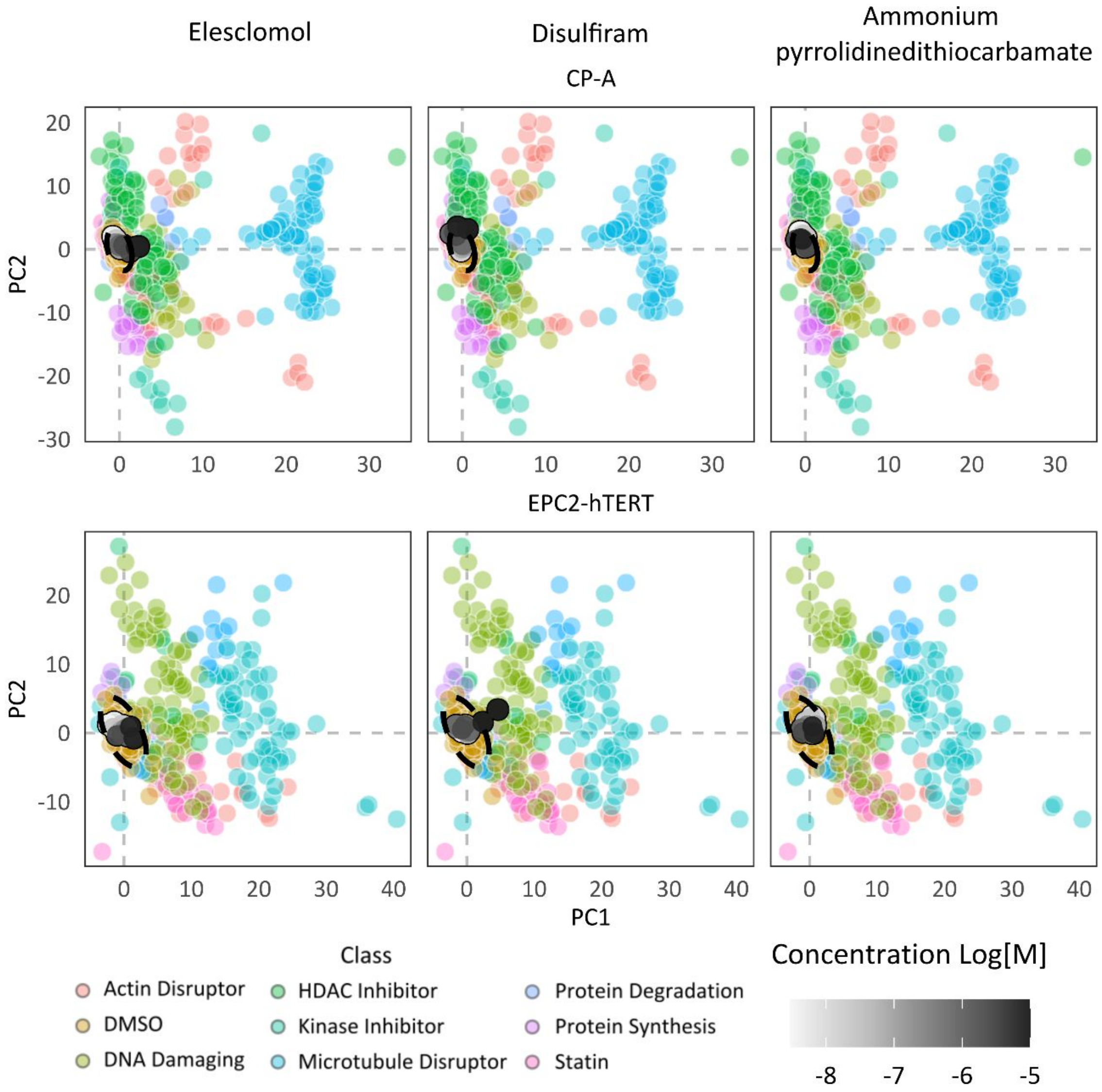

Supplementary Figure 2: Phenotypic dose responses, for elesclomol, disulfiram and ammonium pyrrolidinedithiocarbamate in the tissue-matched control cell lines. Library of reference compounds coloured by mechanistic class. Compound dose response overlay coloured by concentration.

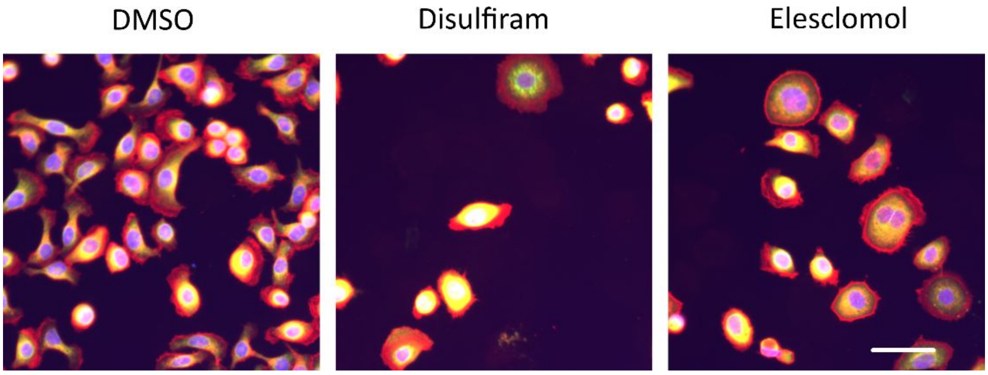

Supplementary Figure 3: Colour combined images for DMSO, disulfiram (100 nM) and elesclomol (3 nM) treatments in the OAC-P4C cell line. Hoescht 33342-nuclei (blue); Phalloidin 594 and Wheat germ agglutinin Alexa Fluor 594- F-actin and golgi and plasma membrane (red); Concanavalin A Alexa Fluor 488- Endoplasmic reticulum (green). Scale bars 50 µm.

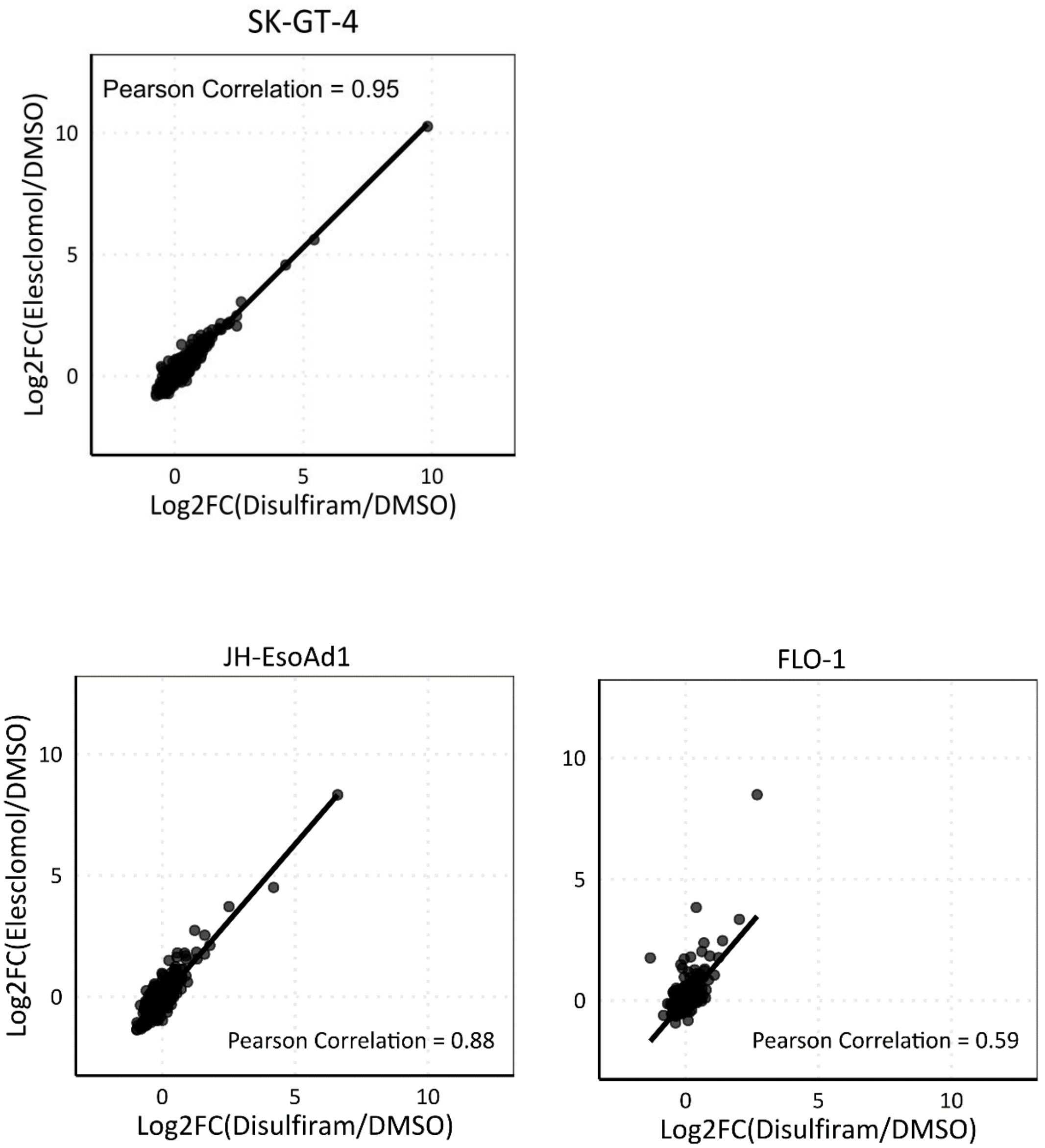

Supplementary Figure 4: Disulfiram and Elesclomol induced log2 fold change gene expression changes in SK-GT-4, JH-EsoAd1 and FLO-1 cell lines.

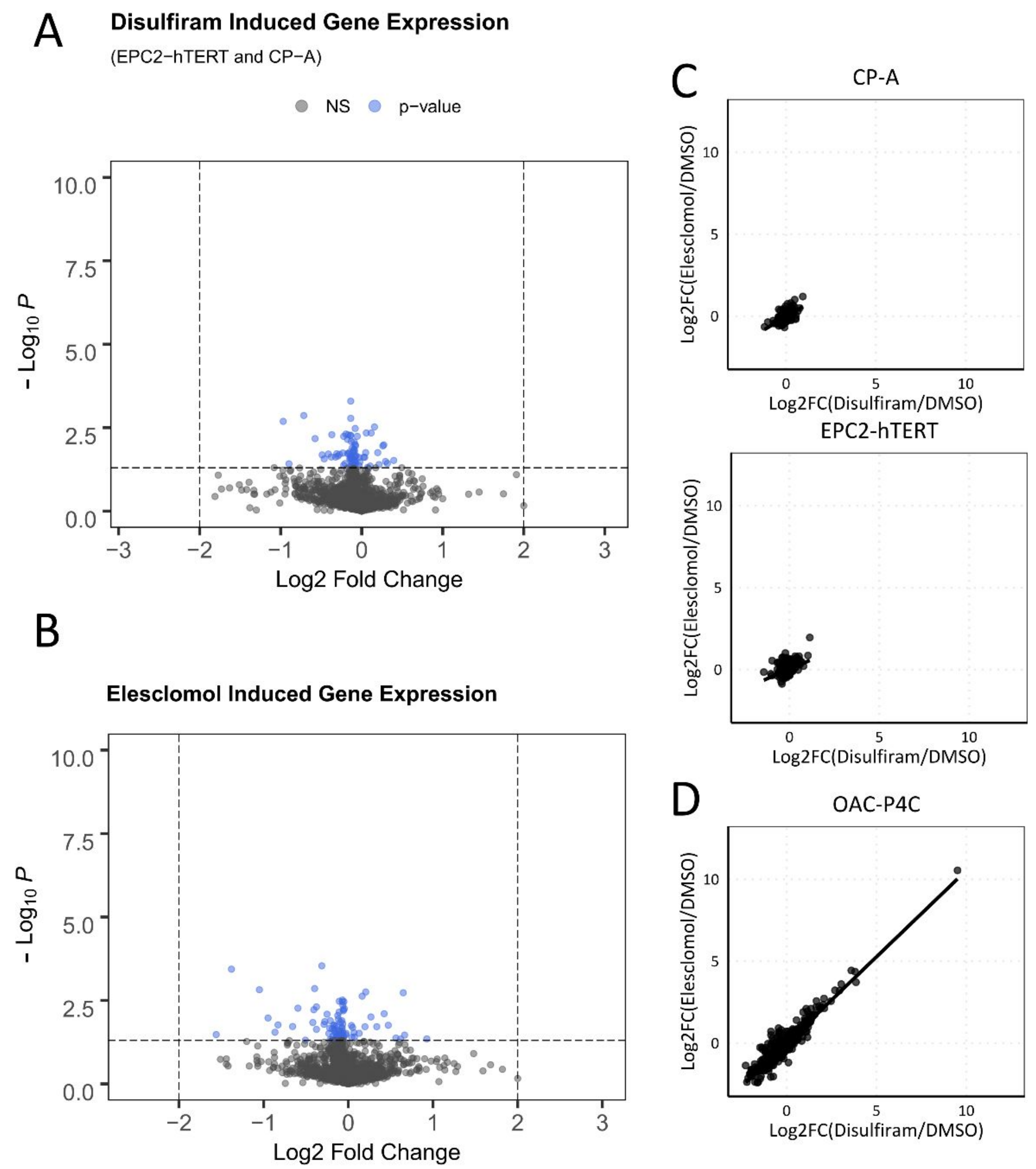

Supplementary Figure 5: Treatment induced gene expression studies in tissue-matched control cell lines. Differential expression analysis for A) Disulfiram, B) Elesclomol, induced gene expression for EPC2-hTERT and CP-A. n = 1. No significant genes after correcting for multiple testing. Correlation plot for Disulfiram and Elesclomol induced log2 fold change gene expression changes in C) tissue-matched controls CP-A and EPC2-hTERT, D) Sensitive OAC cell line OAC-P4C for comparison.

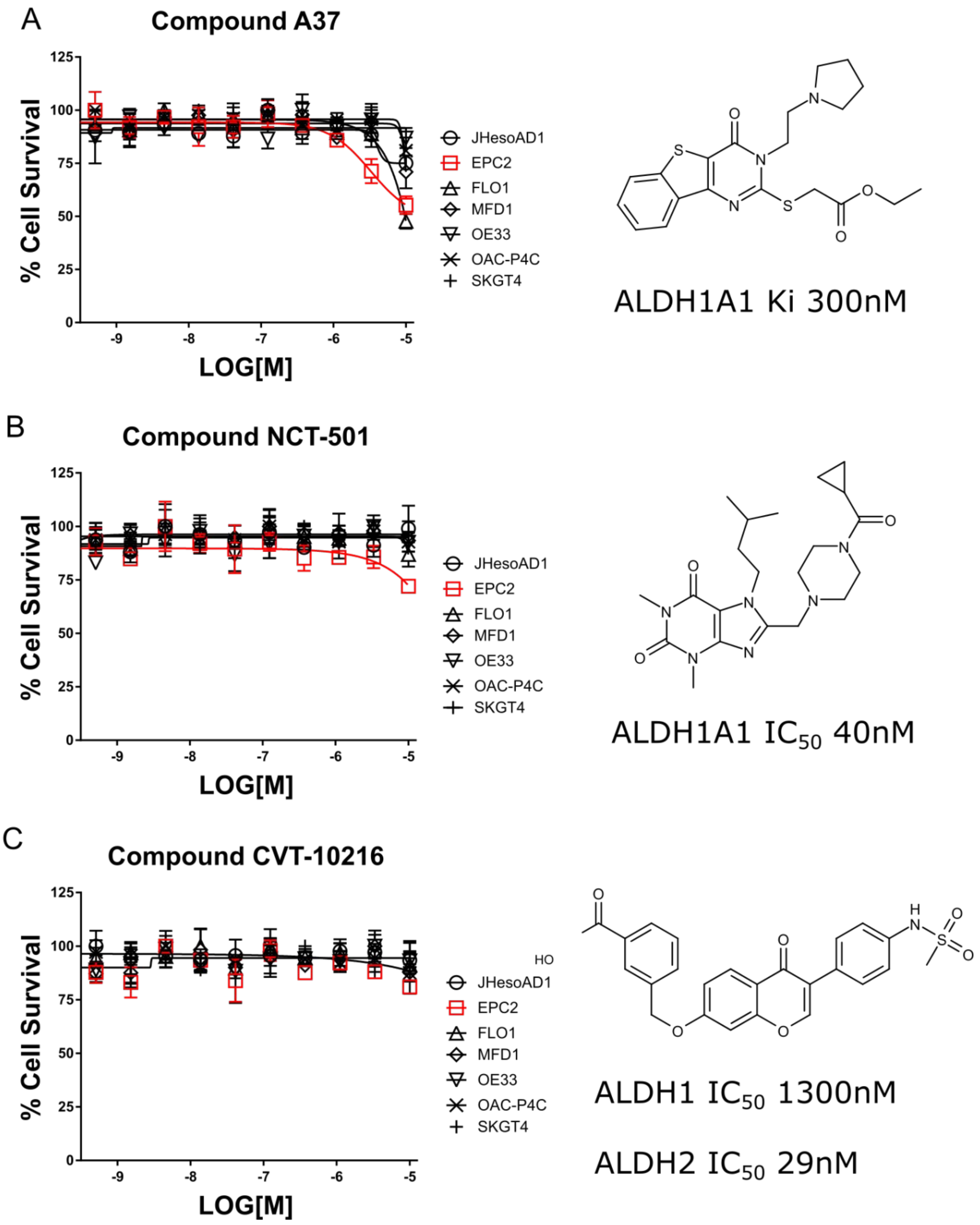

Supplementary Figure 6: Alcohol dehydrogenase inhibitor dose responses. A) A37, B) NCT-501, C) CVT-10216

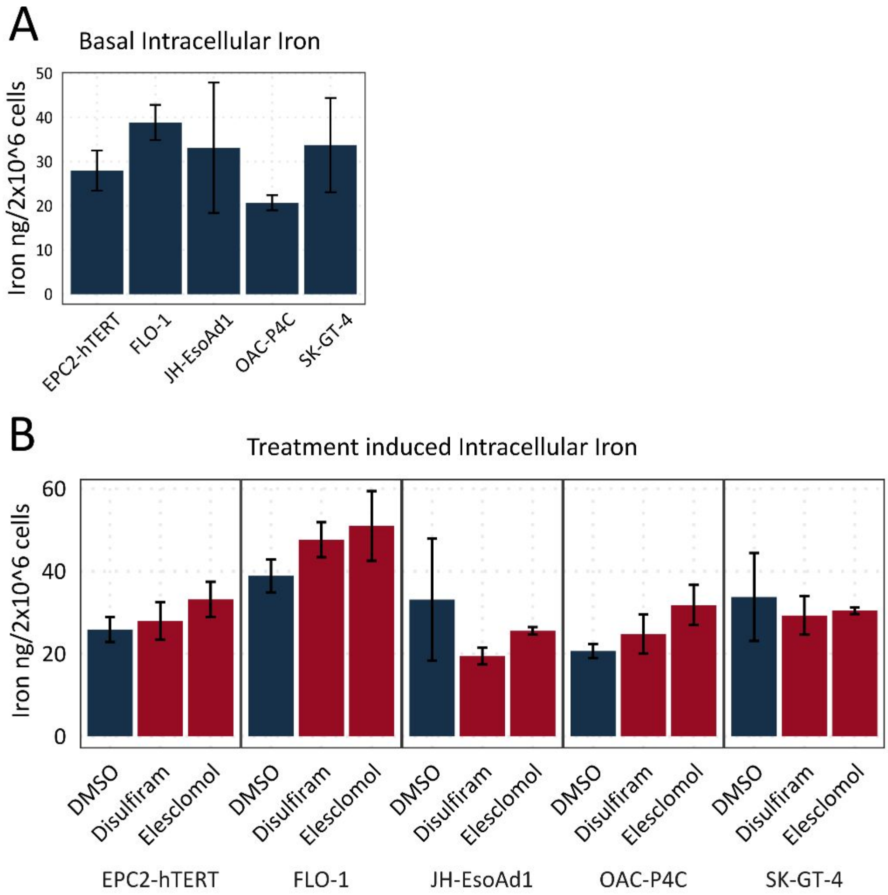

Supplementary Figure 7: Iron ICP-MS. A) Basal and B) Treatment induced intracellular iron levels determined by inductively coupled plasma mass spectrometry. Error bars indicate SE. n= 3.

A

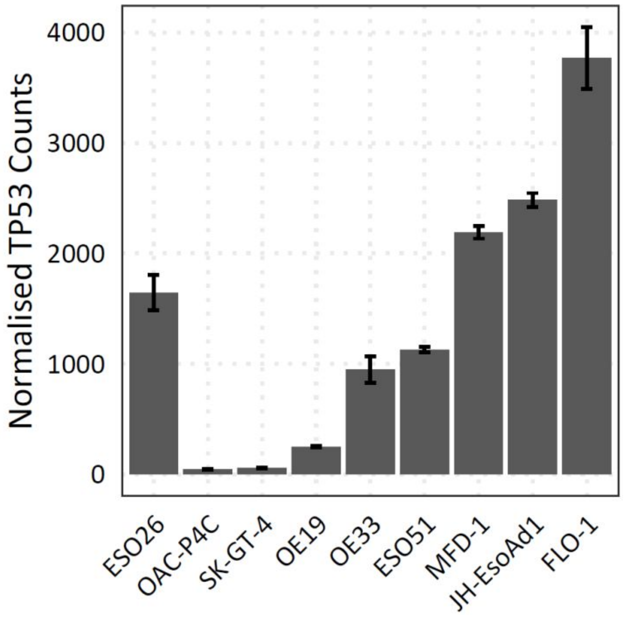

B

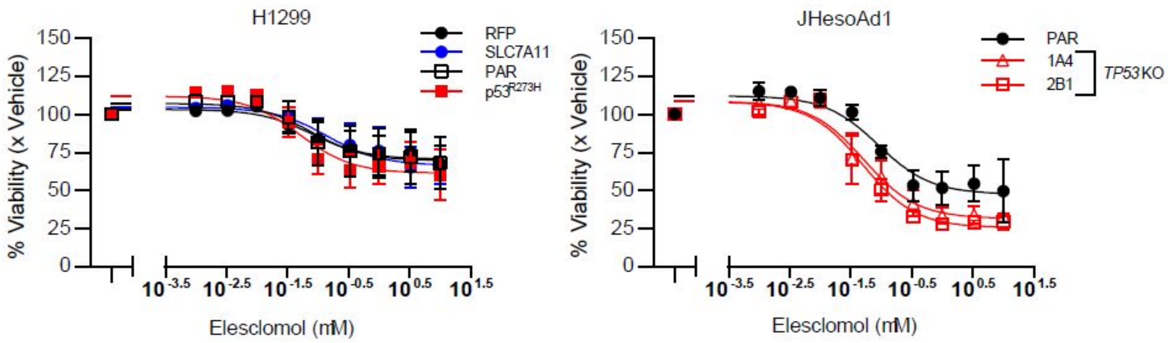

Supplementary Figure 8: TP53. A) TP53 expression levels across OAC cell panel ranked by sensitivity to elesclomol. **B)** Does responses for H1299 p53 WT non-small cell lung cancer line (PAR=parental) and isogenic P53 mutant (R273H) and knockout (RFP) cells. JH-EsoAd1 (p53 mutant) resistant cell line (PAR=parental) and two isogenic TP53 knockout clones.

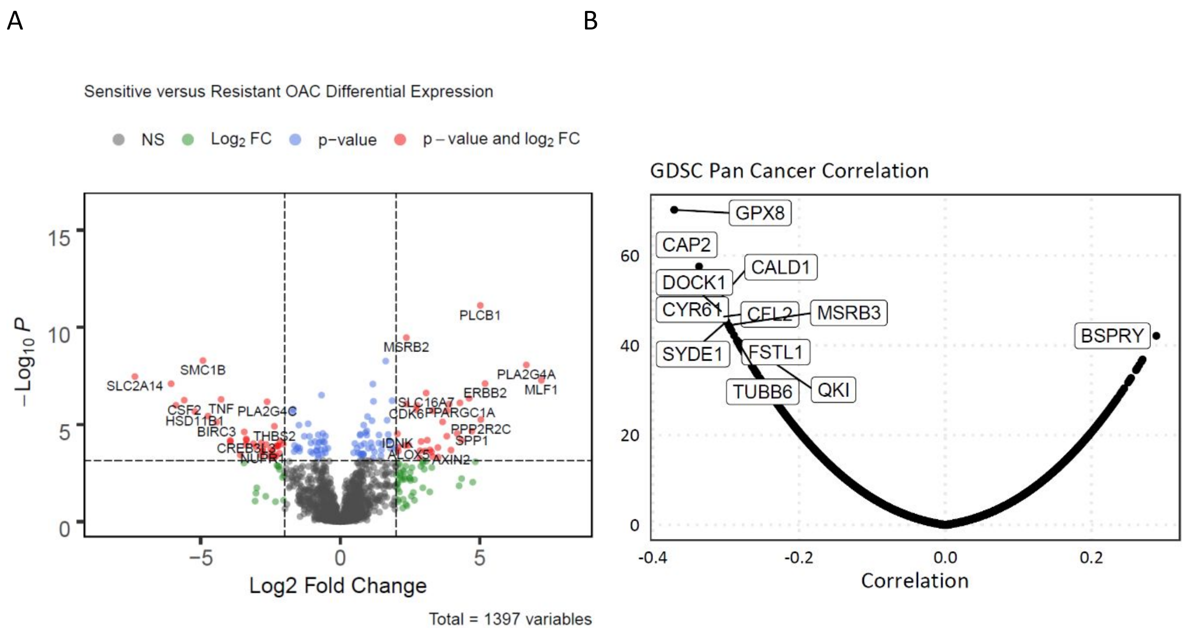

Supplementary Figure 9: Gene expression defining elesclomol sensitivity. A) Differential expression analysis. Sensitive cell lines: OAC-P4C, SK-GT-4, OE19, ESO26. Resistant cell lines: FLO-1, JH-EsoAd1, ESO51. N=3. P value threshold equivalent to adjusted p value 0.05. Gene total = 1397. NanoString nSolver software. B) Correlation analyses of Genomics of Drug Sensitivity in Cancer. RMA normalised expression values for 17,737 genes against elesclomol area under the curve for 916 cell lines.

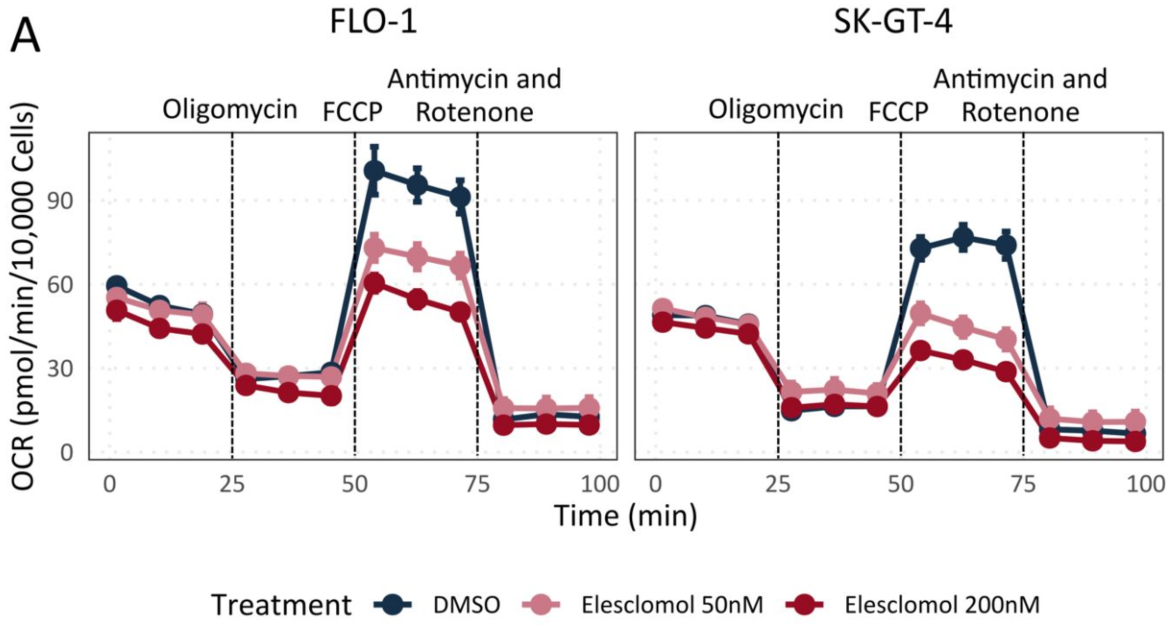

Supplementary figure 10: Effect of elesclomol treatment on mitochondrial function. A) Exemplar oxygen consumption rate (OCR) traces for resistant and sensitive cell lines when treated with varying concentrations of elesclomol for 6 hrs prior to assay.
